# Condensed-phase isomerization through tunnelling gateways

**DOI:** 10.1038/s41586-022-05451-0

**Published:** 2022-10-20

**Authors:** Arnab Choudhury, Jessalyn A. DeVine, Shreya Sinha, Jascha A. Lau, Alexander Kandratsenka, Dirk Schwarzer, Peter Saalfrank, Alec M. Wodtke

**Affiliations:** 1 Institute for Physical Chemistry, University of Goettingen, Goettingen, Germany; 2 Department of Dynamics at Surfaces, Max Planck Institute for Multidisciplinary Sciences, Goettingen, Germany; 3 Department of Chemistry, University of Potsdam, Potsdam, Germany; 4 Present Address: Department of Chemistry, University of California, Berkeley, Berkeley, CA USA

**Keywords:** Chemical physics, Origin of life, Reaction kinetics and dynamics, Reaction mechanisms

## Abstract

Quantum mechanical tunnelling describes transmission of matter waves through a barrier with height larger than the energy of the wave^1^. Tunnelling becomes important when the de Broglie wavelength of the particle exceeds the barrier thickness; because wavelength increases with decreasing mass, lighter particles tunnel more efficiently than heavier ones. However, there exist examples in condensed-phase chemistry where increasing mass leads to increased tunnelling rates^2^. In contrast to the textbook approach, which considers transitions between continuum states, condensed-phase reactions involve transitions between bound states of reactants and products. Here this conceptual distinction is highlighted by experimental measurements of isotopologue-specific tunnelling rates for CO rotational isomerization at an NaCl surface^3,4^, showing nonmonotonic mass dependence. A quantum rate theory of isomerization is developed wherein transitions between sub-barrier reactant and product states occur through interaction with the environment. Tunnelling is fastest for specific pairs of states (gateways), the quantum mechanical details of which lead to enhanced cross-barrier coupling; the energies of these gateways arise nonsystematically, giving an erratic mass dependence. Gateways also accelerate ground-state isomerization, acting as leaky holes through the reaction barrier. This simple model provides a way to account for tunnelling in condensed-phase chemistry, and indicates that heavy-atom tunnelling may be more important than typically assumed.

## Abstract (web summary)

Measurements of  isomerization rates  of CO isotopologues on an NaCl surface show a nonmonotonic mass dependence that arises from  resonantly enhanced cross-barrier coupling, or ‘tunnelling gateways’, which  are intrinsic to condensed-phase tunnelling.

## Main

Although often ignored, tunnelling can dominate the rates of important chemical reactions. For example, tunnelling appears in chemical models of cold interstellar clouds^5,6^ postulated to be the birthplace of the ‘molecules of life’^7^, and is invoked to explain the rates of some important enzyme reactions at physiological temperatures^8–10^. Experts in these fields have established how to experimentally detect condensed-phase tunnelling^11^, where it may be inferred from the influence of temperature *T* on the reaction rate constant *k*(*T*). While over-the-barrier reactions typically obey the Arrhenius relation—

1 $$k(T)=A{{\rm{e}}}^{-{E}_{{\rm{a}}}/{k}_{{\rm{B}}}T},$$

where *A* and *E*_a_ are the Arrhenius prefactor and activation energy, respectively, and *k*_B_ is the Boltzmann constant—tunnelling rates often become nearly *T*-independent at low temperature^12,13^. This ‘deep-tunnelling regime’ reflects tunnelling from the ground state of the reactant. Vibrationally excited states below the barrier may tunnel more rapidly than the ground state; hence, thermally activated tunnelling exhibiting Arrhenius-like *T* dependence is also possible. In this case, tunnelling is distinguishable from an over-the-barrier process by the Arrhenius parameters extracted from fitting experimental data to equation (1); *E*_a_ will be smaller than the expected barrier height, and *A* will be anomalously small, reflecting the low probability of tunnelling through the barrier^11,14^.

Kinetic isotope effects (KIEs) are also used to identify tunnelling, where they are often much larger than those of over-the-barrier reactions. The largest KIEs are found for H-substitution by deuterium (D) or tritium (T), for example, the 80× acceleration seen for H versus D in room-temperature soybean lipoxygenase-1 catalysis^10^. KIEs for heavy-atom tunnelling require low *T* to observe, for example, the 4× acceleration of ^12^C^16^O versus ^13^C^16^O cascading on copper (*T* < 6 K)^12^. The mass dependence in these examples fulfils the expectations of our textbook tunnelling picture. However, in a study of CO binding to myoglobin at 20 K, ^12^C^18^O reacted 20% faster than ^13^C^16^O. This unexpected mass dependence was attributed to ‘structure effects’, although a deeper understanding was not possible at the time^2^.

To better understand condensed-phase tunnelling, one desires an experimentally accessible model system that is simpler than myoglobin or enzymes, as well as a first-principles theory to which the behaviour of the system can be meaningfully compared. Unfortunately, such model systems are rare, and even for relatively simple systems, rigorous quantum treatments can be daunting owing to the many degrees of freedom inherent in the condensed phase. Recently, a simple isomerization reaction was identified that enables a meaningful comparison of experiment to theory. When CO adsorbs at a NaCl(100) surface^15–17^, it may bind with the C atom or the O atom facing the surface, resulting in distinct vibrational signatures for the two orientations^3,18^. Subsequent work on the potential energy surface for this system^19^ found the O-bound isomer lying approximately 610 cm^−1^ higher in energy than the more stable C-bound isomer, with an approximately 570 cm^−1^ barrier for O-bound → C-bound isomerization^4^. Calculation of the two-dimensional (CO rotation *θ*, translation from surface *Z*) wavefunctions residing in the double-well system yielded thousands of vibrational states; those lying below the barrier height are predominantly localized in either the C-bound or O-bound well, providing the basis for a first-principles quantum theory of state-to-state isomerization rates.

In this paper, we report measurements of thermal tunnelling rates for conversion of O-bound to C-bound CO in buried monolayers of ^12^C^16^O, ^13^C^16^O and ^13^C^18^O, obtained using infrared absorption spectroscopy. At *T* ≈ 20 K, the rates vary by a factor of approximately four between isotopologues and depend nonmonotonically on mass; ^13^C^16^O isomerizes faster than ^12^C^16^O, which isomerizes faster than ^13^C^18^O. We present a quantum rate theory for condensed-phase tunnelling that is based on Fermi’s golden rule (FGR), where a phonon bath induces transitions between pairs of vibrational states localized on opposite sides of the isomerization barrier. Through the confluence of the state pairs’ energies, wavefunctions and interactions with the phonon bath, this model provides an intuitive understanding of the erratic mass dependence as arising from accidentally accelerated transitions between specific reactant and product states, which we term tunnelling gateways.

## Experimental isomerization rates

The full experimental procedure is described in Supplementary Information section 1 (Supplementary Fig. 1). Briefly, an isotopically pure layer of CO (^13^C^18^O, ^13^C^16^O or ^12^C^16^O) is dosed with a pulsed molecular beam onto an NaCl(100) surface. This is subsequently buried beneath overlayers of ^12^C^16^O using a second pulsed molecular beam. Laser excitation at *T* = 19 K produces nonequilibrium populations of O-bound CO; the temperature is then set to a chosen reaction temperature (19–24 K). The subsequent return to equilibrium is observed using time-resolved Fourier-transform infrared (FTIR) absorption spectroscopy^3^.

**Fig. 1:**
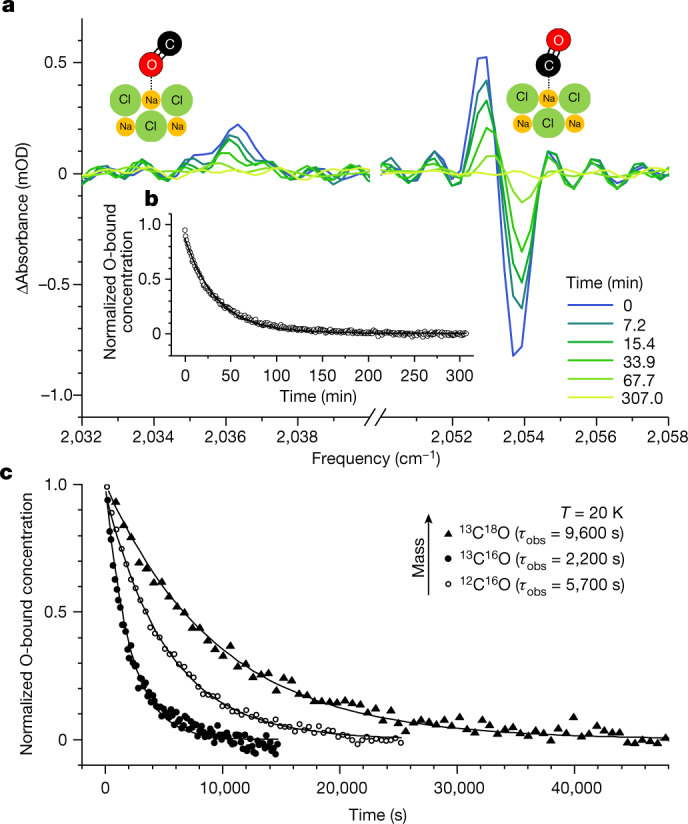
Observed kinetics of CO orientational isomerization. **a**, Absorbance (mOD = 10^−^^3^ log_10_(*I*_0_/*I*), where *I*_0_ and *I* are the intensities of the incident and transmitted beams, respectively) FTIR spectra for a buried ^13^C^18^O monolayer at 21 K following overlayer excitation, where the spectrum of the unexcited sample is subtracted. The lineshape of the approximately 2,053 cm^−1^ feature reflects the time-dependent C-bound centre frequency, which shifts as neighbouring O-bound adsorbates convert to C-bound. Illustrations of the two isomers are shown as inset schematics. **b**, Time-dependent O-bound concentration (open symbols) derived from the spectra in **a** and exponential fit (solid line). **c**, Representative kinetic traces for all three isotopologues. See Supplementary Information section 3 (Supplementary Table 1) for all measured lifetimes and error bars.

Figure 1a shows representative FTIR spectra for a buried ^13^C^18^O monolayer following ^12^C^16^O overlayer excitation^18^. Here a reference pre-excitation absorption spectrum is subtracted from transient post-excitation spectra. The O-bound isomer absorbs at approximately 2,036 cm^−1^ and disappears with time, whereas the depletion feature at approximately 2,053 cm^−1^ is due to C-bound CO and recovers with time. From these features, the time dependence of the O-bound concentration is obtained (Supplementary Information section 2 and Supplementary Figs. 2–7); this is shown in Fig. 1b with an exponential fit that yields the observed characteristic decay time *τ*_obs_ (Supplementary Information section 3 and Supplementary Table 1). Figure 1c shows measured decays at 20 K for all three isotopologues, exhibiting a KIE that is, to our knowledge, the largest ever seen for nonhydrogenic isotopic substitution. Figure 1c also shows a surprising nonmonotonic mass dependence of the reaction rate—^13^C^16^O reacts most rapidly, followed by ^12^C^16^O, then ^13^C^18^O.

**Fig. 2:**
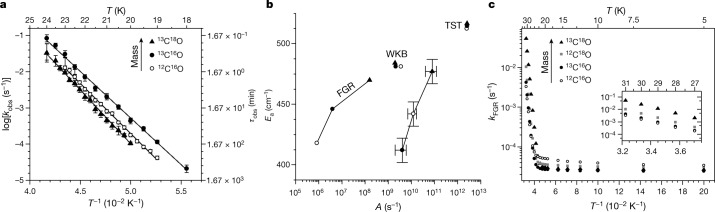
Thermal behaviour of O-bound → C-bound isomerization. **a**, The experimentally derived rate constants for three isotopologues (symbols) and fits based on equation (1) (solid lines) are shown in an Arrhenius plot. Note that *k*_obs_ = 1/*τ*_obs_ and that because *τ*_obs_ varies by more than two orders of magnitude, the fits exhibit little correlation error between the derived values of *A*_obs_ and ${E}_{{\rm{a}}}^{{\rm{obs}}}$. **b**, Arrhenius parameters obtained from experiment (shown with error bars) and all considered theories (TST, transition state theory; WKB, Wentzel–Kramers–Brillouin model; Supplementary Table 2). Results for ^12^C^16^O, ^13^C^16^O and ^13^C^18^O are shown as open circles, filled circles and filled triangles, respectively. **c**, Thermal rates from the FGR model. The temperature dependence of the thermal rate constants for four isotopologues predicted by the FGR model shows a thermally activated (inset) and a deep-tunnelling (*T* < 19 K) regime. The thermally activated tunnelling exhibits a nonintuitive mass dependence similar to that seen in experiment. All experimental results in **a** and **b** represent 95% confidence intervals.

Figure 2a presents experimentally determined temperature-dependent rate constants *k*_obs_ for all three isotopologues. Notably, the nonmonotonic mass dependence of Fig. 1c is seen over the entire range where direct comparison is possible (19–23 K). In addition, the rates of ^13^C^18^O approach those of ^13^C^16^O as temperature increases, suggesting that the rates for ^13^C^18^O might exceed those of both lighter isotopologues above 24 K. Arrhenius behaviour is observed for all three isotopologues, indicated by the linear fits in Fig. 2a; these reflect the strongly isotopologue-dependent Arrhenius prefactors *A*_obs_ and activation energies ${E}_{{\rm{a}}}^{{\rm{obs}}}$ (Fig. 2b; see Supplementary Table 2). We note that these parameters, as do the rate constants, show a nonmonotonic mass dependence.

## Arrhenius parameters indicate tunnelling

Ignoring the peculiar mass dependence, the ${E}_{{\rm{a}}}^{{\rm{obs}}}$ Arrhenius parameters in Fig. 2b provide the first suggestion that these rate constants reflect a tunnelling process, as all three values are substantially lower than the expected barrier height of 574 cm^−1^ (ref. ^4^). The tunnelling hypothesis is further supported by comparison to expectations for an over-the-barrier process, which can be done using transition state theory (TST). Applying TST to the O-bound → C-bound isomerization (Supplementary Information section 4) yields rate constants that exhibit a normal KIE, in contrast to experiment. The TST rates vary by less than 5% with mass, showing a much weaker KIE than observed. Fitting the TST rates to an Arrhenius form yield *A*^TST^ and *E*_a_^TST^ (Fig. 2b). Comparison of these to experimental Arrhenius parameters show the typical hallmarks of condensed-phase tunnelling; ${E}_{{\rm{a}}}^{{\rm{obs}}}$ values are well below those expected for an over-the-barrier process, and *A*_obs_ values are several orders of magnitude smaller than those predicted by TST.

A commonly used tunnelling model—Wentzel, Kramers, Brillouin (WKB)—was then applied to our system (Supplementary Information section 5 and Supplementary Figs. 8, 9). Arrhenius rate parameters obtained with WKB are also shown in Fig. 2b. As does TST, WKB fails to describe our observations—it predicts a normal mass dependence (that is, lighter isotopologues tunnel faster), and the WKB activation energies do not greatly vary with mass. Overall, WKB predicts a much weaker mass dependence than is seen experimentally.

## Quantum gateway hypothesis

We pause here to emphasize the key observations that require explanation: (1) the derived values of ${E}_{{\rm{a}}}^{{\rm{obs}}}$ are substantially below what is expected for an over-the-barrier process, and show a nonmonotonic mass dependence; (2) the derived values of *A*_obs_ are extraordinarily small relative to *A*_TST_ and show a nonmonotonic mass dependence that is correlated with the mass dependence of ${E}_{{\rm{a}}}^{{\rm{obs}}}$; and (3) the KIE is large with a seemingly erratic mass dependence, but when expressed in Arrhenius form, exhibits a strong correlation between the prefactor and activation energy. These observations can be understood by introducing the concept of tunnelling gateways.

In CO rotational isomerization, we envision a tunnelling gateway as consisting of a thermally populated vibrational quantum state of the O-bound isomer that couples, through collisions with its environment, to a vibrationally excited C-bound state localized across the barrier. Particularly for high excitation of hindered rotation, these O-bound and C-bound wavefunctions extend into the classically forbidden region, leading to nonzero coupling. Interactions (that is, collisions) with the environment may then transfer population from the O-bound state to the C-bound state. We note that the two states in the gateway can be nondegenerate, as the environment may, within limits, compensate for the energy difference by accepting or emitting a phonon.

The gateway concept qualitatively explains the erratic mass dependence, because isotopic substitution produces energy shifts large enough to make different gateways important in different isotopologues. The gateway concept also allows us to interpret the observed *T* dependence by providing an intuitive understanding of the Arrhenius parameters in Fig. 2b. In this picture, the energy of the initial gateway state in the O-bound well is approximately ${E}_{{\rm{a}}}^{{\rm{obs}}}$; the tunnelling rate constant is then given by the thermal population of the gateway, $\exp (-{E}_{{\rm{a}}}^{{\rm{obs}}}/{k}_{{\rm{B}}}T)$, and the gateway’s intrinsic propensity to tunnel, *A*_obs_. With this in mind, a simple pattern emerges—*A*_obs_ increases with increasing ${E}_{{\rm{a}}}^{{\rm{obs}}}$ owing to the rapid increase in tunnelling probability as the gateway’s energy comes closer to the top of the barrier. The fact that we can systematize the KIE in this way qualitatively supports the gateway tunnelling hypothesis.

## Quantum rate theory for isomerization

To test the gateway hypothesis more rigorously, we developed a quantum rate theory of condensed-phase tunnelling based on FGR (Supplementary Fig. 10). Here we solve the time-independent Schrödinger equation to obtain all vibrational eigenstates up to the classical isomerization barrier for each isotopologue, using the previously reported potential energy surface^4^. These states define a two-dimensional ‘system’ in perturbative contact with a ‘bath’ of phonon states, produced by low-frequency motions of the CO monolayer and the NaCl solid. We then compute thermal rates for population transfer between all O-bound localized states to all C-bound localized states using FGR to describe the phonon-bath-induced perturbative coupling between system states. For further details, see Supplementary Information section 6 (Supplementary Figs. 11–22 and Supplementary Table 3).

Figure 2c shows the FGR rate constants for four isotopologues—those studied experimentally and ^12^C^18^O—for *T* = 5–31 K. Two regimes are clearly seen: thermally activated tunnelling from 27–31 K exhibiting Arrhenius behaviour (Fig. 2c, inset), and deep tunnelling between 5–19 K. Although it does not show perfect agreement, the FGR model does capture some of the peculiarities observed experimentally. As with experiment, there is a range of temperatures where heavier isotopologues tunnel faster than lighter ones. The extracted FGR Arrhenius parameters are correlated in a similar way as ${A}_{{\rm{obs}}}/{E}_{{\rm{a}}}^{{\rm{obs}}}$ (see Fig. 2b), and show a strong mass dependence: *A*_FGR_ increases with mass by more than two orders of magnitude, and ${E}_{{\rm{a}}}^{{\rm{FGR}}}$ increases with mass by approximately 12%. By contrast, WKB prefactors decrease by a factor of less than two, and WKB activation energies are insensitive to mass.

**Fig. 3:**
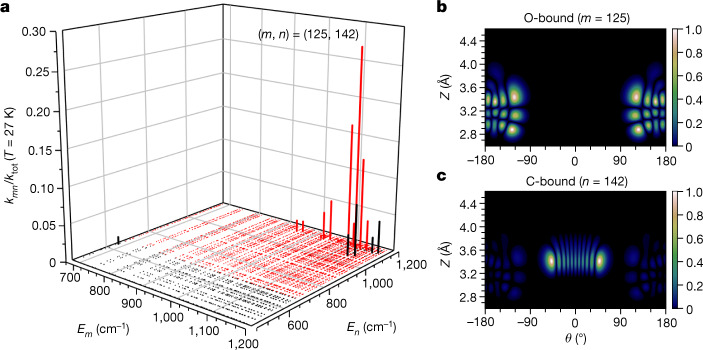
Gateway picture arising from the FGR model. **a**, Visualization of the state-to-state FGR rate constants for ^13^C^18^O shows that the majority of state pairs do not contribute to the total thermal rate at *T* = 27 K. Each vertical bar indicates the fraction of the total FGR rate constant represented by the state-to-state rate *k*_*mn*_, describing transition from an O-bound state *m* at energy *E*_*m*_ to a C-bound state *n* at energy *E*_*n*_. Red lines indicate upward transitions (*E*_*n*_ > *E*_*m*_) and black lines indicate downward transitions (*E*_*n*_ < *E*_*m*_). **b**,**c**, Normalized wavefunction amplitudes of the dominant gateway for ^13^C^18^O thermally activated tunnelling at 27 K. Panel **b** shows the reactant (O-bound) state and panel **c** shows the product (C-bound) state located 71 cm^−1^ below the barrier. Notice the small amount of delocalization exhibited by the acceptor state that enhances the tunnelling rate of the heavy isotopologue.

Figure 3a shows thousands of computed state-to-state rate constants for ^13^C^18^O represented as vertical lines; see Supplementary Fig. 12 for similar plots for ^13^C^16^O and ^12^C^16^O. Each line represents the relative contribution of one state-to-state FGR rate constant to the total thermal rate at 27 K. For all isotopologues, thermally activated tunnelling is dominated by population transfer between a few pairs of vibrationally excited states, located close enough in energy to be coupled by absorption or emission of a single phonon. This accounts for the large amount of empty space in Fig. 3a. The vibrational wavefunctions involved in the dominant ^13^C^18^O tunnelling gateway are shown in Fig. 3b,c (those of ^12^C^16^O and ^13^C^16^O are provided in Supplementary Fig. 15); these wavefunctions possess considerable excitation of hindered rotation, as reflected in their nodal structure along the *θ* coordinate. Because they arise through accidental resonances (when accounting for phonon absorption or emission), these gateways appear at different energies for each isotopologue, giving rise to strongly mass-dependent activation energies in the FGR model that lead to unexpected KIEs.

Below 19 K, the FGR rate constants are nearly temperature-independent, as only the O-bound ground state is populated (see Table 1). In this deep-tunnelling regime, the FGR model again predicts an unexpected KIE. It is interesting to note that the predicted ground-state FGR tunnelling rates are reduced by only three orders of magnitude compared to the thermally activated regime. The state-to-state rate constants (Supplementary Fig. 16) show again that this is due to tunnelling gateways acting as leaky holes through the barrier, as the total rate is dominated by population transfer to a small number of C-bound states. In Table 1, one can see that the FGR rate of ground-state tunnelling exceeds the WKB predictions by several orders of magnitude; furthermore, ground-state WKB rates appear with the normal mass dependence and are smaller than FGR rates by 13–15 orders of magnitude (*k*_WKB-MEP_) and by 10–12 orders of magnitude when assuming a corner-cutting path (*k*_WKB-CC_). This suggests that WKB estimations may underestimate the importance of tunnelling in chemical reactions.

**Table 1:** Predicted tunnelling rate constants for ground-state CO rotational isomerization on NaCl(100)

	ZPE^a^ (meV)	*k*_WKB-MEP_^b^ (s^−1^)	*k*_WKB-CC_^c^ (s^−1^)	*k*_FGR_ (s^−1^)	*k*_obs_ (s^−1^)
^**12**^**C**^**16**^**O**	3.76	3.7 × 10^−18^	4.2 × 10^−15^	3.3 × 10^−5^	
^**13**^**C**^**16**^**O**	3.69	5.2 × 10^−19^	6.8 × 10^−16^	2.4 × 10^−5^	
^**13**^**C**^**18**^**O**	3.60	6.8 × 10^−20^	1.2 × 10^−16^	2.6 × 10^−5^	3.0 × 10^−8d^

^a^The one-dimensional zero-point energy (ZPE) along the *θ* coordinate. ^b^Calculated for the minimum energy path between reactant and product (Supplementary Information section 5). ^c^Calculated along a corner-cutting pathway where the tunnelling distance was minimized (Supplementary Information section 5). ^d^Upper limit for a buried monolayer.

We caution that our FGR model predicts a transition temperature to the deep-tunnelling regime at higher temperature than in experiment—only thermally activated tunnelling is observed experimentally, indicating that the onset of the true deep-tunnelling regime is below 19 K. Correspondingly, the FGR ground-state tunnelling rate is higher than the experimental estimation (Supplementary Information section 7 and Supplementary Fig. 23). As outlined in Supplementary Information section 6, this is due to the fact that in the FGR model, a nonburied system with inherently larger tunnelling probabilities was considered. Despite this caveat, it is clear that the FGR model (Fig. 2c) can produce a very different temperature dependence with enhanced rates for deep tunnelling compared to WKB (Supplementary Fig. 9).

## Concluding remarks

As the reader may have noted, the FGR model does not perfectly reproduce all details of the experimental data. In fact, owing to the accidental nature of tunnelling gateways and the simplicity of our model (see Supplementary Information section 6.a.v), an excellent replication of experiment would itself be accidental. The presented model aims not to numerically reproduce experimental rate constants, but to give a qualitative explanation for a nonmonotonic mass dependence of tunnelling rates. In this respect, the theory is highly successful, and allows for a deeper understanding of observed thermal effects. Most importantly, it provides a more rigorous basis for tunnelling gateways. The gateway hypothesis, which was synthesized purely from experimental observations, was not built into the FGR model. That these gateways appear from our simplified approach (see Fig. 3a) is the critical theoretical result of this work, as it shows that the quantum mechanics underlying condensed-phase isomerization leads to a different conceptual picture of tunnelling than is typically considered.

Although the observations and ideas of this work may appear unprecedented, we point out that the unusual isotope effect seen in this work is reminiscent of reaction resonances seen in gas-phase hydrogen-transfer reactions. For the F + H_2_ → HF + F and F + HD → HF + D reactions, tunnelling to quasibound states on the product side of the reaction barrier enhance reactivity and influence the product-scattering angular distributions^20^. Moreover, isotopic substitution changes which resonances are important, dramatically altering the reactivity. We also see similarities with other work in the condensed phase—specifically, the leaky holes through the reaction barrier predicted by the FGR model are similar to observations of the resonant tunnelling of electrons^21–27^.

Past theories of thermally activated tunnelling in enzymes employed a semi-empirical picture resembling the Marcus theory of electron transfer, where fluctuating solvent modes produce tunnelling resonances between reactant and product^28,29^. Thermal activation arises from protein conformational changes that produce a ‘tunnelling-ready state^30^’, leading to a reduction in barrier height or tunnelling distance^10^. Our observations reveal a nuanced variation of this picture; condensed-phase tunnelling occurs when environmental interactions induce transitions between bound states localized across the reaction barrier. Here states are not brought into degeneracy by thermal fluctuations; rather, energy defects are compensated for by exchange of phonons.

Fortuitously, tunnelling through quantum gateways is clearly demonstrated in this uniquely informative CO/NaCl(100) system. Nevertheless, we see no reason why such a model could not be extended to other systems. Indeed, it is possible that such a gateway picture could explain the KIEs of a previous work^2^ that studied CO binding to myoglobin.

We note in closing that the conclusions of this work may be of particular relevance to interstellar chemistry. Long thought to be dominated by barrierless gas-phase ion–molecule reactions^31^, it has more recently become apparent that reactions with barriers may take place by tunnelling in ices deposited on dust grains^32^. Current interstellar chemical models may overlook facile tunnelling reactions, because the importance of quantum gateways has not yet been recognized.

## Online content

Any methods, additional references, Nature Portfolio reporting summaries, source data, extended data, supplementary information, acknowledgements, peer review information; details of author contributions and competing interests; and statements of data and code availability are available at 10.1038/s41586-022-05451-0.

## Supplementary information

Supplementary Information This file contains Supplementary Methods (section 1), Supplementary Text (sections 2–7), Supplementary Figures 1–23, Supplementary Tables 1–3 and Supplementary References.

## Data Availability

All data are available in the text or the Supplementary Information.

## Code availability

Eigenstate and rate calculations reported in this paper were performed with codes available at https://github.com/sinhas95/Condensed-phase-isomerization.
