# Supplementary material for: Condensed-phase isomerization through tunnelling gateways
Source: Nature. 2022 Oct 20;612(7941):691–5. doi: 10.1038/s41586-022-05451-0 (PMC9771804; doi:10.1038/s41586-022-05451-0)
Supplement: Supplementary file 1 — This file contains Supplementary Methods (section 1), Supplementary Text (sections 2–7), Supplementary Figures 1–23, Supplementary Tables 1–3 and Supplementary References. [file 41586_2022_5451_MOESM1_ESM.pdf]

nature portfolio

https://doi.org/10.1038/s41586-022-05451-0

Supplementary information

Condensed-phase isomerization through tunnelling gateways

In the format provided by the authors and unedited

SUPPORTING INFORMATION FOR

Condensed phase isomerization through tunneling gateways

Authors: Arnab Choudhury[1,2], Jessalyn A. DeVine[2], Shreya Sinha[3], Jascha A. Lau[1,2,a], Alexander Kandratsenka[2], Dirk Schwarzer[2], Peter Saalfrank[3] and Alec M. Wodtke[1,2]*

Affiliations:

[1]Institute for Physical Chemistry, University of Goettingen; Goettingen, Germany.

[2]Dept. of Dynamics at Surfaces, Max-Planck-Institute for Multidisciplinary Sciences; Goettingen, Germany.

[3]Dept. of Chemistry, University of Potsdam, Potsdam, Germany

[a]Current address: Department of Chemistry, University of California, Berkeley, California 94720, USA

*Corresponding author. Email: alec.wodtke@mpinat.mpg.de.

S1. Experimental Methods 2
S2. Data Analysis 3
a. Baseline correction 4
b. Relative O-Down Concentrations 4
c. Exciton Model 4
S3. Table of Lifetimes 8
S4. Transition State Theory 9
S5. WKB Theory 10
S6. Quantum Rate Theory: One-phonon Assisted Thermal Rates 11
a. Model and FGR Rate Expressions 11
i. Total Hamiltonian 11
ii. System Hamiltonian and Eigenstates 12
iii. Bath Hamiltonian and System-Bath Coupling 14
iv. Fermi's Golden Rule Rate Expressions 16
v. Limitations of the FGR Model 19
b. Results 20
i. Arrhenius Parameters 20
ii. Importance of Tunneling Gateways 21
iii. Convergence of System Wavefunctions and Ground State Tunneling Rate 22
S7. Attempted Measurement of the Ground State Tunneling Lifetime for $^{13}C^{18}O$ 23
S8. Supplementary Figures 23
S9. References 37

S1. Experimental Methods

Figure S1 shows a schematic diagram of the experimental apparatus used in the current work. The molecular beam dosing procedure used for sample preparation has been described previously.[1] Briefly, a fixed number of molecular beam pulses of CO gas ($^{12}C^{16}O$: Sigma-Aldrich; 99.99% ^{12}C and 99.95% ^{16}O, 99.9% chemical purity, $^{13}C^{18}O$: Sigma-Aldrich; 99.9% ^{13}C and 99.2% ^{18}O, 99.8% chemical purity and $^{13}C^{16}O$: Sigma-Aldrich; 99.07% ^{13}C and 97.2% ^{16}O, 99.99%) were directed onto a UHV-cleaved NaCl(100) crystal surface held at 25K to form the isotopically pure monolayer. After monolayer dosing, samples were cooled to 22K and ~100 layers of $^{12}C^{16}O$ were added. Cryogenic temperatures were achieved by coupling the sample to a dual-stage helium cryo-cooler (RDK-408D2, Sumitomo), and the sample was held inside a liquid nitrogen-cooled cold shield during measurements to limit warming due to room-temperature surfaces in the laboratory. The sample temperature was monitored and controlled using a Model 335 Lakeshore cryogenic temperature control unit. Sample characterization was performed using a Fourier Transform Infrared (FTIR) spectrometer (VERTEX 70V, Bruker) operating in external mode using either an InSb or an MCT detector cooled by liquid nitrogen.

Following sample preparation and characterization, the sample was cooled to 19K and a pulsed infrared laser fixed at 2138.6 cm^{-1} was used to induce the $v = 0$ to $v = 1$ vibrational excitation in the $^{12}C^{16}O$ overlayer[2] and the vibrational energy was efficiently transported to the the $^{13}C^{16}O$ or $^{13}C^{18}O$ monolayer.[3] For the $^{12}C^{16}O$ monolayer buried beneath a $^{12}C^{16}O$ overlayer, vibrational energy transport is not possible; here, the monolayer was pumped directly with IR laser light at 2152.5 cm^{-1}. A voltage pulse-activated shutter controlled the number of laser shots used to pump the sample. The laser was then blocked and the temperature was raised to the value of interest. The time dependent absorption spectra were then observed using a Bruker FTIR spectrometer equipped with the Rapid-Scan functionality, the light source of which was aligned to be collinear with the excitation laser. From these measurements, the sample absorption spectrum was obtained as a function of time. Changes in the spectrum were determined by using the absorption spectrum of the unexcited sample as the reference channel. These experiments were performed for three different monolayer isotopologues as well as various surface temperatures. The infrared light used

to excite the sample was generated by a difference frequency mixing setup wherein the output of a tuneable dye laser pumped by the second harmonic of a seeded 10 Hz Nd:YAG laser mixes with the 1064nm Nd:YAG fundamental light in a $LiIO_3$ crystal.

To ensure that there was no influence of exposure to the FTIR light source on the resultant decay of O-down population, a set of two experiments were carried out where FTIR spectra were acquired at ~90 second intervals following excitation of the sample. In one set of experiments, it was ensured that the glow bar was only illuminating the sample during spectral acquisition (~30 seconds per spectrum). Thus, the effective exposure to the FTIR light source was reduced to ¼ of that characteristic for the other measurement, where the FTIR light source was always irradiating the sample. As shown in Fig. S2 the two kinetic traces are indistinguishable, demonstrating that exposure to the glow bar does not influence the measured lifetimes of O-down molecules.

S2. Data Analysis

It is obvious from Fig. S4 that the integral over the absolute value of the C-down feature (area B + C) is considerably larger than area C – B and hence produces kinetic traces with larger signal-to-noise ratios. In the following section we will show that area B + C (method 3) is in fact proportional to the O-down concentration, and analyzing the time dependence from the kinetic traces using B + C gives more reliable time constants than methods 1 and 2, in particular when the rate of flipping is very high (e.g. at $T > 23\text{K}$) or the laser excitation efficiency is low (e.g., $^{12}C^{16}O$ monolayer samples).

In this section we describe in detail how FTIR absorbance spectra are used to obtain the time dependent concentrations of O-down CO on NaCl(100). We first perform a baseline correction (section S2a). We then consider how to extract from the spectral data the peak integrals that are related to the O-down population at each time step (section S2b), and relate the obtained peak areas to O-down population using an exciton model (section S2c).

a. Baseline correction

The baseline of the obtained absorbance spectra shifts over the course of a lifetime measurement which can last many hours, hence a baseline correction must be implemented. To achieve this, a straight line was fitted through the baseline of spectra obtained at different times in the measured decay and subtracted. Figure S3 shows examples of two experimentally obtained spectra without (black) and with (orange) this baseline correction.

b. Relative O-Down Concentrations

The relative fraction of O-down molecules in the monolayer can be obtained from the difference absorbance spectrum (see Fig. S4) by determining the area A under the O-down peak (method 1). It can also be calculated from the decrease in the C-down fraction relative to the unexcited sample by integrating the whole C-down feature (method 2). Since its shape arises from a drop in absorption intensity as well as a broadening and red-shift of the band, the integral of the difference spectrum consists of a positive (area B) and a negative (area C) contribution and is given by the area C – B. These two methods give essentially the same result (A ≈ C – B), as the decreased C-down fraction is equal to the increased O-down fraction.

c. Exciton Model

It is necessary to show that the absorbance of CO molecules in the sample is linearly proportional to their population. To do this, we simulate absorbance spectra for a simplified matrix of CO molecules with the help of a well-known exciton model.[4,5] The monolayer is taken to have a 1×1 unit cell, where CO monomers are either aligned vertically upward (C-down) or downward (O-down). The position of O-down molecules were chosen randomly. From the simulated spectra (Fig. S5) we determine the correlation between the fraction of C-down molecules and the integrated absorbances calculated by the three methods discussed above. We show that for O-down fractions generated in our experiments, all three methods give integrals which are linear in the O-down population (Fig. S6). We find that the integrated absorbances obtained by method 3 discussed above give better signal-to-noise in the kinetic traces (Fig. S7).

The monolayer is considered to be a collection of N molecules, where the ground and first-excited vibrational state of isolated molecule i are given by ϕ_i and ϕ_i', respectively, where the relative energy of

the first excited state is given by $hc\tilde{\nu}_0$. The zeroth-order collective *N*-molecule states are then perturbed by their pairwise dipole-dipole interactions.

In the ground state, the collective zeroth-order wavefunction is given by

$$\psi_0 = \phi_1\phi_2\phi_3 \dots \phi_N. \tag{1}$$

The collective single-excitation wavefunctions, where a single adsorbate is in its first excited state, form an *N*-fold degenerate sub-space with energy $hc\tilde{\nu}_0$ (in the case that all molecules have the same orientation), so that the lth single excitation exciton wavefunction is given by

$$\psi_l = C_{l1}\phi_1'\phi_2\phi_3\cdots\phi_N + C_{l2}\phi_1\phi_2'\phi_3\cdots\phi_N + C_{l3}\phi_1\phi_2\phi_3'\cdots\phi_N + \cdots$$
$$+C_{li}\phi_1\phi_2\phi_3\cdots\phi_i'\cdots\phi_N + \cdots + C_{lN}\phi_1\phi_2\phi_3\cdots\phi_N' \tag{2}$$

where $l = 1, 2, 3, \dots, N$. The ith component in (2) then corresponds to the zeroth-order single excitation state where molecule *i* is in its first vibrational excited state indicated by a prime.

To determine the true (coupled) excitonic wavefunctions and eigenvalues, we must diagonalize the perturbation Hamiltonian in the basis consisting of the individual terms included in (1) and (2); i.e., ψ_0 and the *N* single-excitation ψ_l, where $C_{li} = \delta_{li}$ is the Kronecker delta. Neglecting electrostatic interactions beyond electric dipole-dipole, the perturbation Hamiltonian is written as

$$H^{(1)} = \sum_{i=1}^{N}\sum_{j>1}^{N} u_i u_j f_{ij} \qquad \text{where} \tag{3}$$

$$f_{ij} = \frac{1}{4\pi\epsilon_0 R_{ij}^3}\left[\boldsymbol{e}_i \cdot \boldsymbol{e}_j - 3(\boldsymbol{e}_i \cdot \boldsymbol{r}_{ij})(\boldsymbol{e}_j \cdot \boldsymbol{r}_{ij})\right]. \tag{4}$$

Here, u_i and $\boldsymbol{e}_i$ are the magnitude and unit vector of the dipole moment of molecule i, respectively, $\boldsymbol{r}_{ij}$ is the unit vector from molecule i to molecule j, and R_{ij} is the intermolecular distance. For a polar diatomic molecule, the dipole moment is parallel to the bond axis of the molecule, so $\boldsymbol{e}_i$ also indicates the orientation of the molecule i.

The diagonal element of the perturbation matrix associated with the 0th vibrational ground state (1) is given by

$$H_G^{(1)} = \langle \psi_0 | H^{(1)} | \psi_0 \rangle = \mu^{00} \mu^{00} \sum_{i=1}^{N} \sum_{j<i}^{N} f_{ij} \ , \tag{5}$$

and those associated with each excited state *k* represented in (2) may be written as

$$H_{kk}^{(1)} = \langle \psi_k | H^{(1)} | \psi_k \rangle = \mu^{00} \mu^{00} \sum_{i=1, i\neq k}^{N} \sum_{j<i, j\neq k}^{N} f_{ij} + \mu^{00} \mu^{11} \sum_{i=1, i\neq k}^{N} f_{ik} \tag{6}$$

$$= H_G^{(1)} + \Delta H_{kk}^{(1)} \quad \text{with} \quad \Delta H_{kk}^{(1)} = \frac{(\mu^{11} - \mu^{00})\mu^{00}}{hc} \sum_{i=, i\neq k}^{N} f_{ik} \, . \tag{7}$$

Here, μ^{00} (-0.112 Debye) and μ^{11} (-0.087 Debye)[4] are the permanent dipole moments of a CO molecule in vibrational state $v = 0$ and $v = 1$, respectively. The off-diagonal elements of the perturbation are given by

$$H_{ij}^{(1)} = \langle \psi_i | H^{(1)} | \psi_j \rangle = \mu^{01} \mu^{01} \, f_{ij} \tag{8}$$

where μ^{01} is $v = 0 \rightarrow 1$ transition dipole moment (0.105 Debye).[4]

We now apply *N*-fold degenerate perturbation theory to calculate the first-order energy corrections $E_l^{(1)}$,

$$\sum_{l=1}^{N} \left[H_{\alpha\beta}^{(1)} - E_l^{(1)} \delta_{\alpha\beta} \right] = 0, \tag{9}$$

where $H_{\alpha\beta}^{(1)} = \langle \psi_\beta | H^{(1)} | \psi_\alpha \rangle \ (\alpha, \beta = 1, \cdots N)$ are the matrix elements from Eqs. (6) through (8), and $\delta_{\alpha\beta}$ is the Kronecker delta. Diagonalization of $H^{(1)}$ gives *N* eigenvalues, $E_l^{(1)} = \Delta\tilde{\nu}^{(l)} hc$, as well as the associated eigenvectors Ψ_l, whose total energy is given by $E_l = hc(\tilde{\nu}_0 + \Delta\tilde{\nu}^{(l)}) = hc\tilde{\nu}^{(l)}$. We note that this applies to the case where all adsorbates have the same orientation. For a monolayer with *m* flipped molecules, the same derivations apply, but there are now two degenerate sub-spaces; the (*N* – *m*)-dimensional C-down subspace, and the *m*-dimensional O-down subspace.

The integrated absorption cross-section for excitation of the monolayer to the lth single-excitation exciton state obtained from the diagonalization discussed above is given by

$$\bar{\sigma}^{(l)} = \frac{8\pi^3 \tilde{\nu}^{(l)}}{Nhc} \left| \sum_{j=1}^{N} \langle \Psi_l | \boldsymbol{\mu}_j | \psi_0 \rangle \right|^2 . \tag{10}$$

The absorption spectrum of chosen monolayer geometry $S(\tilde{\nu})$ was then calculated as a Gaussian convolution of the discrete stick spectrum $\left[\Delta\tilde{\nu}^{(l)}, \bar{\sigma}^{(l)}\right]$ associated with the N coupled single-excitation exciton states. Simulations were performed using a 30×30 grid of CO molecules; increasing the grid size was not found to substantially change the resultant spectra. Resulting difference absorbance spectra, $\Delta S(\tilde{\nu}) = S(\tilde{\nu}) - S_0(\tilde{\nu})$ where $S_0(\tilde{\nu})$ is the spectrum for the 100% C-down monolayer, are shown for various O-down coverages in Fig. S5.

From Fig. S5, we can determine the relationship between integrated absorbances and the percentage of molecules flipped in the monolayer, by calculating

$$A_1 = \int_{2030\text{cm}^{-1}}^{2040\text{cm}^{-1}} \Delta S(\tilde{\nu})\, d\tilde{\nu} \qquad \text{(method 1)}$$

$$A_2 = -\int_{2050\text{cm}^{-1}}^{2055\text{cm}^{-1}} \Delta S(\tilde{\nu})\, d\tilde{\nu} \qquad \text{(method 2)}$$

$$A_3 = \int_{2050\text{cm}^{-1}}^{2055\text{cm}^{-1}} |\Delta S(\tilde{\nu})|\, d\tilde{\nu} \qquad \text{(method 3)}$$

for grids with varying fractional O-down populations. The results of applying the three methods to the simulated spectra are shown in Fig. S6A.

Lifetime measurements were limited to samples where not more than ≈30% of the molecules were flipped. An example of a typical lifetime measurement experiment is shown in Fig. S6B, where we can clearly see that the O-down peak area (I_O) is ∼0.13 fraction of the full monolayer (I_C^0), indicating that ~30% of the molecules were flipped. The integrated absorbances calculated from method 3 were then used to determine the concentration of O-down molecules for the reasons discussed above.

S3. Table of Lifetimes

Table S1 provides the flipping lifetimes obtained for all samples studied in this work. The lifetimes are obtained by fitting the O-down population data, calculated as described in the previous section, to a single exponential decay and then averaging the resultant fit parameters over several experiments. The uncertainties are estimated with 95% confidence interval. Rates were fit to an Arrhenius model to extract the parameters given in Table S2.

Table S1. Flipping lifetimes τ_{obs} for all CO monolayer isotopologues across all temperatures studied. Temperatures could be reproduced within ± 0.003 K; the absolute accuracy is estimated to be better than ± 0.05 K.

T (K)	Decay Constant (s)		
	$^{13}C^{18}O$	$^{13}C^{16}O$	$^{12}C^{16}O$
18.00	-	48000 ± 10000	-
19.00	-	8700 ± 1000	24000 ± 2400
19.25	-	-	15700 ± 1600
19.50	-	3700 ± 400	12000 ± 1200
19.75	-	-	8400 ± 800
20.00	9600 ± 600	2300 ± 500	5700 ± 600
20.25	5700 ± 600	-	3500 ± 350
20.50	4200 ± 120	930 ± 100	2550 ± 250
20.75	3100 ± 600	-	1440 ± 140
21.00	2200 ± 350	490 ± 60	1240 ± 120
21.25	1500 ± 300	-	788 ± 80
21.50	1100 ± 250	286 ± 40	620 ± 70
21.75	600 ± 100	-	422 ± 40
22.00	500 ± 100	138 ± 20	306 ± 30
22.25	310 ± 50	-	198 ± 20
22.50	210 ± 35	74 ± 12	139 ± 14
22.75	170 ± 20	-	93 ± 13
23.00	106 ± 6	33 ± 7	78 ± 8
23.25	80 ± 12	-	-
23.50	50 ± 15	19 ± 3	-
24.00	30 ± 15	12 ± 3	-

Table S2: Arrhenius rate parameters for isotopically-selected CO flipping on NaCl(100) in a buried monolayer. All A-factors are in units of s^{-1} and all E_a-values are in units of cm^{-1}.

	Experiment*		TST#		FGR Rate Theory†		WKB CC**		WKB MEP††	
	A_{obs}	E_a^{obs}	A_{TST}	E_a^{TST}	A_{FGR}	E_a^{FGR}	A_{WKB}	E_a^{WKB}	A_{WKB}	E_a^{WKB}
$^{12}C^{16}O$	$1.2\pm0.5\times10^{10}$	442 ± 10	2.50×10^{12}	512.5	8.2×10^{5}	418 ± 8	5.3×10^{8}	463	3.6×10^{9}	481
$^{13}C^{16}O$	$4\pm2\times10^{9}$	412 ± 10	2.55×10^{12}	514.5	3.9×10^{6}	446 ± 4	3.5×10^{8}	465	2.1×10^{9}	481
$^{13}C^{18}O$	$8\pm4\times10^{10}$	477 ± 10	2.59×10^{12}	516.6	1.6×10^{8}	470 ± 1	3.0×10^{8}	468	2.0×10^{9}	484

*Obtained by performing an error-weighted fitting of Eq. (1) to the measured rate-constants. Error bars reflect the 95% confidence intervals.

#Found by fitting the predictions of transition state theory to an Arrhenius expression; see section S4. For the TST calculations we used the harmonic frequencies of the full dimensional potential (i.e. 6 modes for the O-bound CO and 5 modes for the TS) given in ref. 6.

†Calculated for $26.8 < T < 31\ K$. See section S6.

**Obtained using a corner cutting tunneling path as described in section S5.

††Obtained using the minimum energy tunneling path as described in section S5.

S4. Transition State Theory

The rate constant for the conversion of O-bound to C-bound CO as a function of temperature can be obtained using transition state theory (TST),

$$k(T) = \frac{k_B T}{h} \frac{Q^{\ddagger}}{Q_r} e^{-E_0/k_B T} \tag{11}$$

where E_0 is the classical barrier to reaction from a previously reported two-dimensional DFT calculation for a coverage of ¼ monolayer.[6] (See also Sec. S6 below.) The partition functions of the transition state and the O-down reactants, $Q^{\ddagger}$ and Q_r, are obtained by treating all motion (internal stretch, frustrated rotation, and frustrated translation) as simple harmonic oscillators,

$$Q^{\ddagger} = \prod_{i=1}^{5} \frac{1}{1 - \exp\left(-\frac{h\nu_i^{\ddagger}}{k_B T}\right)} \qquad Q_r = \prod_{i=1}^{6} \frac{1}{1 - \exp\left(-\frac{h\nu_i^{r}}{k_B T}\right)}$$

where frequencies $\nu_i^{\ddagger}$ and ν_i^{r} were obtained from Ref. 6 for the $^{12}C^{16}O$ isotope of CO on the NaCl(100) surface for a coverage of ¼ monolayer. Note that the products include all real-frequency modes. For the other isotopologues, the harmonic frequencies were scaled using the appropriate reduced mass μ, i.e.

$$\nu_{i,2} = \nu_{i,1} \sqrt{\frac{\mu_1}{\mu_2}} \; . \tag{12}$$

Putting this all together, we obtain activation energies E_a^{TST} and prefactors A_{TST} by fitting the 19-24 K TST predictions of the rate constants to an Arrhenius form. E_a^{TST} and A_{TST} are temperature independent over the range relevant to this work and are isotope specific. See table S2. The isotope effect on the derived lifetimes (given by $1/k$) shows a small and monotonic increase in lifetime with mass. Specifically, at 20K we see a 15% increase in the TST lifetime in going from $^{13}C^{16}O$ to $^{13}C^{18}O$. The isotope effect decreases to 12% at 24K. Similar results were seen when comparing $^{13}C^{16}O$ to $^{12}C^{16}O$.

S5. WKB Theory

A commonly employed model used to consider tunneling through a barrier is the semi-classical Wentzel-Kramers-Brillouin (WKB) approximation. The tunneling rate in the WKB in mass-weighted coordinates is defined by the following equation

$$k_{WKB}(E) = \nu \exp\left[-\frac{2\sqrt{2}}{\hbar}\int_{s_1}^{s_2} ds\,\sqrt{V(s)-E}\right] = \nu \exp\left[\int_{\theta_1}^{\theta_2} d\theta\,\sqrt{2\left[M\left(\frac{dZ}{d\theta}\right)^2 + \mu r_{\mathrm{eq}}^2\right][V(\theta,Z)-E]}\right]. \quad (13)$$

Here, ν is the attempt frequency taken here to be the frequency of the frustrated rotation, $V(s)$ is the potential energy along a reaction coordinate s; the integration limits s_1 and s_2 are the turning points at energy E on each side of the barrier. Note that we have used the RHS of Eq. 13 where the Z coordinate is parametrized w.r.t the θ coordinate (see Fig. S8 (a)). Here, $M = M_{\mathrm{C}} + M_{\mathrm{O}}$ and $\mu = M_{\mathrm{C}}M_{\mathrm{O}}/M$ are the CO total and reduced mass, respectively, and $r_{\mathrm{eq}} \approx 1.14$Å is the CO bond equilibrium distance. Using the 2D PES from Ref. [6], we calculated the ground-state tunneling rate $k_{WKB}(E_{ZPE})$, where E_{ZPE} is the 1D zero-point energy of the O-bound isomer. We emphasize that we have used the one-dimensional ZPE of the frustrated rotation mode associated with the lowest (O-bound) eigenstates. Hence, we assume that the ZPE associated with the Z-coordinate is not effective at promoting isomerization. We performed the WKB integrals numerically along the minimum energy path (MEP) and along a corner cutting pathway[7] shown in Fig. S8; both of these pathways were extracted from the same 2D PES mentioned above. From the ZPE level we could easily calculate s_1 and s_2. We performed the WKB calculations for four isotopologues, $^{12}C^{16}O$, $^{13}C^{16}O$, $^{13}C^{18}O$, $^{12}C^{18}O$, the former three used in the experimental studies, both in the WKB model as well as the Fermi's Golden Rule (FGR) model, described later in Section S6.

We also used the WKB model to simulate thermally activated tunneling from 5 to 31 K on the same PES used above. This relies on a thermal average of the state specific WKB tunneling rate constants $k_{WKB}(E_m)$.

$$k_{tot}^{WKB}(T) = \sum_m k_m^{WKB}(T) = \sum_m \frac{e^{-\beta(E_m-E_0)}}{\sum_l e^{-\beta(E_l-E_0)}} k_{WKB}(E_m) \quad (14)$$

All vibrational eigenstates *m* dominantly localized in the "O-bound" well, up to the classical isomerization barrier, were included in the WKB thermal averaging. In this way we include the influence of vibration on the WKB tunneling rate. The results are shown in Fig. S9. The isotope-dependent thermal WKB rate constants were fit to an Arrhenius form—those fitted parameters are reported in Table S2.

S6. Quantum Rate Theory: One-phonon Assisted Thermal Rates

Fermi's Golden Rule (FGR) combined with system-bath approaches is a widely used formulation for the study of vibrational relaxation dynamics in condensed-phase systems, where FGR gives a transition rate between a chosen initial and final state of the system. This rate depends on the coupling between the two states as well as the density of final states. Here, we use such a system-bath approach to develop an FGR model of isomerization rates for CO at the NaCl (100) surface; here, the "system" is defined by the "O-bound" / "C-bound" initial/final states of the CO molecule on the NaCl (100) surface, and the "bath" corresponds to the phonons of the CO/NaCl interface (i.e. motion of the NaCl surface atoms plus the neighboring CO molecules). System and bath modes are coupled by a vibration-phonon coupling operator which enters the FGR rate expressions as specified below. The basic physics is illustrated in Fig. S10.

Below, we describe our system, the chosen initial and final states, and the coupling model in more detail (section S6a). Specific results beyond what was presented in the main text are described in section S6b. Rates were calculated for four isotopologues of CO ($^{12}C^{16}O$, $^{13}C^{16}O$, $^{12}C^{18}O$, and $^{13}C^{18}O$); however, the in-depth analysis of the results provided below is only shown for those isotopologues which were observed in experiment. Total rates for the fourth isotopologue ($^{12}C^{18}O$) are shown in Figure 2c of the main text.

a. Model and FGR Rate Expressions

i. Total Hamiltonian

In our system-bath approach, our system (an inverting CO molecule on a NaCl (100) surface) is treated in two dimensions (2D), considering the important vibrational adsorbate modes involved in the isomerization reaction, namely θ and Z, which are coordinates for the frustrated rotation and perpendicular

CO-surface motion, respectively. The system modes are coupled to a harmonic bath comprising the NaCl (100) surface phonons as well as the low frequency vibrational modes of the neighboring CO adsorbates up to the Debye frequency of the surface, i.e., 222 cm^{-1}. The model Hamiltonian of the total system is then expressed as

$$\hat{H} = \hat{H}_S + \hat{H}_B + \hat{H}_{SB} \tag{15}$$

where $\hat{H}_S = \hat{H}_S^{2D}$ is the 2D adsorbate Hamiltonian, $\hat{H}_B$ describes the phonon bath, and $\hat{H}_{SB}$ the coupling between adsorbate (system) and bath.

ii. System Hamiltonian and Eigenstates

The 2D system Hamiltonian is given by

$$\hat{H}_S^{2D} = -\frac{\hbar^2}{2M}\frac{\partial^2}{\partial Z^2} - \frac{\hbar^2}{2I}\frac{\partial^2}{\partial \theta^2} + V_{2D}(Z,\theta;r,\phi,X,Y) \tag{16}$$

where M and $I = \mu r_{CO}^2$ are the total CO mass and moment of inertia, respectively, and fixed coordinates are indicated in the potential, V. The definitions of these fixed coordinates can be found in Ref. [6].

As mentioned earlier, this 2D Hamiltonian describes the perpendicular CO-surface motion and frustrated CO rotation for an unburied layer of CO on a NaCl(100) surface. For the potential, $V(Z,\theta)$, we adopt a function which was calculated from gradient-corrected density functional theory (DFT) with dispersion corrections for a coverage of ¼ ML as obtained in Ref. [6]. Details about the anharmonic potential are described in that reference. In particular, one finds a separation of "O-bound" and "C-bound" configurations of about 613 cm^{-1}, with a classical barrier for "O-bound" to "C-bound" reaction of 574 cm^{-1}. Note that for the experimental coverage (one ML, buried under an overlayer), slightly different potential parameters are expected, an effect which we neglect in the present theoretical model.

In Ref. [6], corresponding 2D system eigenfunctions (ψ) were calculated by diagonalizing the system Hamiltonian,

$$\hat{H}_S \psi_k(Z,\theta) = E_k \psi_k(Z,\theta) \tag{17}$$

where $k = m$ ($k = n$) will be used to represent "O-bound" ("C-bound") localized eigenstates. Up to energies very close to the barrier, all eigenstates are either localized in the "O-bound" or "C-bound" well.

These 2D system eigenstates correspond to hindered rotation and CO-surface stretching vibration levels, which when excited, will either relax (downward transitions) or become further excited (upward transitions) via system-bath coupling. Our model accounts for only transitions occurring from “O-bound” to “C-bound” vibrational states, i.e., transitions between states m and n in different wells of the double-well potential, while transitions within the same well are neglected. All eigenstates corresponding to either “O-bound” or “C-bound” configurations below the classical barrier for the isomerization reaction have been considered for the rate calculations (see table S3 below). These eigenstates have been computed for all three CO isotopologues studied in experiment as well as for $^{12}C^{18}O$.

Table S3. Number of initial (N_m, “O-bound”) and final states (N_n, “C-bound”) used for (↑) upward and (↓) downward rates in this work, for different isotopologues.

	$^{12}C^{16}O$	$^{13}C^{16}O$	$^{13}C^{18}O$
$N_m^{\uparrow}$	60	62	65
$N_n^{\uparrow}$	68	72	75
$N_m^{\downarrow}$	60	62	65
$N_n^{\downarrow}$	80	84	89

Since the transition rates in our model are sensitive to the system eigenfunctions, we found it necessary to increase the grid size for the system DOFs compared to Ref. [6] when obtaining the 2D system eigenfunctions in order to obtain converged rates. To extend our calculations to higher grid sizes than possible with ordinary direct-product grids, we utilized a Potential Optimized Fourier Grid Hamiltonian (PO-FGH) method, in analogy to a Potential Optimized Discrete Variable Representation method (PO-DVR)[8] for obtaining the eigenfunctions. In the PO-DVR(FGH) methods, one diagonalizes convenient one-dimensional Hamiltonians using 1D reference potentials along chosen degrees of freedom and uses the obtained eigenfunctions as a zero-order product basis for solving the multidimensional problem. This drastically reduces the size of the multidimensional Hamiltonian to be diagonalized, and one can then transform back from the zero-order product basis to the primitive DVR (FGH) basis used initially for the reference 1D calculations. The modified Hamiltonian now is:

$$\hat{H} = (\hat{T}_Z + V_{ref}^{Z}) + (\hat{T}_\theta + V_{ref}^{\theta}) + V_{2D}(Z,\theta) - V_{ref}^{Z} - V_{ref}^{\theta} \equiv \hat{H}_{1D}^{Z} + \hat{H}_{1D}^{\theta} + \Delta V \quad (18)$$

And the 1D reference potentials are chosen such that:

$$V_{ref}^{Z} = V_{2D}(Z, \min[V_{2D}(Z,\theta);\theta]) \quad (19)$$

$$V_{ref}^{\theta} = V_{2D}(Z_{min}, \theta) \tag{20}$$

Here $\min[V_{2D}(Z,\theta);\theta]$ represents the value of θ which minimizes the 2D potential for a given value of Z. Further, Z_{min} is the Z-value of the minimum of the 2D potential in the "C-bound" configuration (Z_{min} ≈ 3.33 Å). The 1D zero-order states are then given by:

$$\hat{H}_{1D}^{Z}\psi_l(Z) = E_l^z\psi_l(Z) \tag{21}$$

$$\hat{H}_{1D}^{\theta}\psi_p(\theta) = E_p^{\theta}\psi_p(\theta) \tag{22}$$

The 1D eigenvalue problems can be solved with the FGH method using large, spatially uniform grids.[9] To ensure orthonormality of the eigenfunctions, a Gram-Schmidt reorthogonalization routine is also performed on the final 2D eigenfunctions. Further, the number of zero-order product states are chosen such that the total energy does not exceed certain energy cutoff values. Specifically, we selected only those 1D zero-order states of the Z coordinate whose energies were below the desorption limit (~ 1400 cm^{-1}) and the total energy ($E_Z + E_\theta$) was limited to 4000 cm^{-1}. Details concerning convergence with respect to grid sizes will be discussed below.

iii. Bath Hamiltonian and System-Bath Coupling

Next, the bath Hamiltonian is formulated as a sum of N uncoupled harmonic oscillators,

$$\hat{H}_B = \sum_{b}^{N}\left(-\frac{\hbar^2}{2}\frac{d^2}{dq_b^2} + \frac{1}{2}\omega_b^2 q_b^2\right) \tag{23}$$

where $q_b = \sqrt{m_b}x_b$ are mass-weighted coordinates of the bath modes (which need, like masses m_b and Cartesian coordinates x_b, not be further specified in what follows), and ω_b is the harmonic frequency of mode b. The bath Hamiltonian gives rise to a product of uncoupled bath oscillator wavefunctions, $\chi_1(q_1)\chi_2(q_2)\ldots\chi_N(q_N)$, which also need not to be specified further (but will indirectly enter the rate expressions later).

We assume a bilinear system-bath interaction, where coupling is linear in system modes Z, θ and in bath modes, q_b, which allows only for single-phonon excitations / relaxations in the bath. Then, the system-bath coupling Hamiltonian can be written as

$$\hat{H}_{SB} = \sum_{b=1}^{N} \lambda_b(Z,\theta) q_b \,. \tag{24}$$

Here, $\lambda_b(Z,\theta)$ are system-bath coupling functions, taken to be linear in both system modes,

$$\lambda_b(Z,\theta) = c_b^Z (Z - Z_0) + c_b^\theta r_{eq}(\theta - \theta_0), \tag{25}$$

whereby coefficients $c_b^{Z/\theta}$ represent the coupling constants of the corresponding degrees of freedom (DoFs) to the bath modes and r_{eq} is the equilibrium CO bond length (1.141 Å). Z_0 and θ_0 are reference coordinates which, again, need not be specified because they have no influence on the matrix elements entering the rate expressions below.

The coupling constants $c_b^{Z/\theta}$ could, in principle, be calculated from multi-dimensional system-bath potentials and Taylor expansion to first order[10]. Instead, we make two simple approximations. First, we assume that both system modes couple equally to the phonon modes, i.e., we set $c_b^Z = c_b^\theta = c_b$. Following a well-known general procedure,[11] c_b can be obtained from the so-called bath spectral density, $J(\omega_b)$, in a discretized form

$$c_b = \sqrt{\frac{2}{\pi} J(\omega_b)\omega_b \Delta\omega} \,. \tag{26}$$

Here, $\omega_b = b\Delta\omega = b\omega_f/N$ are now equally spaced ($\Delta\omega$) frequencies of the phonon bath up to a cutoff frequency ω_f, taken to be the Debye frequency of the NaCl(100) surface, 222 cm^{-1}. The second simplifying approximation assumes the bath spectral density is proportional to the vibrational density of states (VDOS), ρ_{VDOS}

$$J(\omega_b) = \varepsilon \rho_{VDOS}(\omega_b), \tag{27}$$

where ε is a proportionality constant.

The parameter ε serves to scale the thermal rates to be approximately within the range of experimental values. It is worth noting that since ε appears as a multiplicative factor in the FGR rate expression given below, it has no influence on the kinetic isotope effect.

The VDOS is evaluated for a monolayer of CO molecules on a NaCl (100) surface, with one "O-bound" CO (see Fig. S11a) from *ab initio* molecular dynamics (AIMD) calculations at 30 K, using periodic DFT with the Perdew-Becke-Ernzerhof (PBE) functional and Grimme's D2 dispersion correction Specifically, a 2√2×2√2×3 slab model was used with four CO molecules per unit cell, and only the uppermost NaCl layer and the CO molecules were allowed to move in the AIMD calculations. The VDOS is then extracted from the velocity-velocity autocorrelation function following an approach similar to ref. [12]. The contribution from one "O-bound" CO per unit cell is then projected out and we are left with the VDOS contribution coming only from the NaCl phonons and vibrations of neighboring CO molecules. The resulting VDOS, up to the Debye frequency of the NaCl surface, is shown in Fig. S11b.

iv. Fermi's Golden Rule Rate Expressions

We utilize the FGR formulation for transition rates

$$k_{if} = \frac{2\pi}{\hbar}\left|\langle\Phi_i|\hat{H}_{SB}|\Phi_f\rangle\right|^2 \delta(E_f - E_i) \tag{28}$$

where, initial and final states, Φ_i and Φ_f respectively, are products of the anharmonic system states ψ_m (for i) or ψ_n (for f), and N uncoupled initial and final harmonic bath states. The $\Phi_{i/f}$ are thus eigenstates of the (uncoupled) system-bath Hamiltonian ($\hat{H}_S + \hat{H}_B$) with E_i and E_f being the corresponding initial and final total eigenenergies.

When we plug in the system-bath coupling Hamiltonian from Eq. (24), integrate out the bath modes, and sum over the final bath states, we obtain the one-phonon upward (↑) and downward (↓) rates between pairs of initial "O-bound" states $|m\rangle$ and final "C-bound" states $|n\rangle$ as given in Eqs. (29) and (30)[13] where we use $|m\rangle$ and $|n\rangle$ to denote $|\psi_m\rangle$ and $|\psi_n\rangle$, respectively.

$$k_{mn}^{\uparrow} = \pi \sum_{b}^{N} |\langle n|\lambda_b(Z,\theta)|m\rangle|^2 \frac{\langle n_b\rangle}{\omega_b} \delta(E_m - E_n + \hbar\omega_b) \tag{29}$$

$$k_{mn}^{\downarrow} = \pi \sum_{b}^{N} |\langle n|\lambda_b(Z,\theta)|m\rangle|^2 \frac{\langle n_b\rangle + 1}{\omega_b} \delta(E_m - E_n - \hbar\omega_b) \tag{30}$$

Here, the coupling matrix elements are given by

$$\langle n|\lambda_b(Z,\theta)|m\rangle = \iint \psi_n^*(Z,\theta)\lambda_b(Z,\theta)\psi_m(Z,\theta)dZd\theta. \quad (31)$$

The "upward" ("downward") rates lead to the system being excited (relaxed) from an "O-bound" state with a lower (higher) energy, E_m, to a "C-bound" state with higher (lower) energy, E_n, following absorption (emission) of a single phonon of frequency ω_b, cf. Fig. S10. The Bose-Einstein factors, $\langle n_b\rangle = 1/[\exp(\hbar\omega_b/k_BT)-1]$, give the thermally-averaged quantum number of the b-th phonon mode.

Taking everything together, and making use of $\delta(x-x_0)=\delta(x_0-x)$ and $\delta[a(x-x_0)]=\frac{1}{|a|}\delta(x-x_0)$ we can rewrite Eqs. (29) and (30) as

$$k_{mn}^{\uparrow}(T) = \frac{2\varepsilon}{\hbar}|V_{mn}|^2\sum_{\mathrm{b}}\rho(\omega_b)\langle n_b\rangle\Delta\omega\delta(\omega_b-\omega_{nm}) \quad (32)$$

with $\omega_{nm} = (E_n - E_m)/\hbar > 0$ and,

$$k_{mn}^{\downarrow}(T) = \frac{2\varepsilon}{\hbar}|V_{mn}|^2\sum_{\mathrm{b}}\rho(\omega_b)\langle n_b+1\rangle\Delta\omega\delta(\omega_b-\omega_{mn}) \quad (33)$$

with $\omega_{mn} = (E_m - E_n)/\hbar > 0$. Here, $V_{mn} = \langle m|(Z-Z_0)+r_{eq}(\theta-\theta_0)|n\rangle$ is the matrix element connecting "O-bound" state $|m\rangle$ with "C-bound" state $|n\rangle$.

In the following rate expressions, we use a continuum limit,

$$\lim_{N\to\infty,\Delta\omega\to 0}\sum_{b}^{N} f(\omega_b)\delta(\omega_b-\omega_0)\Delta\omega = \int_{-\infty}^{+\infty} f(\omega)\delta(\omega-\omega_0)d\omega = f(\omega_0)$$

such that the FGR rates take the form

$$k_{mn}^{\uparrow}(T) = \frac{2\varepsilon}{\hbar}|V_{mn}|^2\rho(\omega_{nm})\langle n_{\omega=\omega_{nm}}\rangle \quad (34)$$

$$k_{mn}^{\downarrow}(T) = \frac{2\varepsilon}{\hbar}|V_{mn}|^2\rho(\omega_{mn})\big(\langle n_{\omega=\omega_{mn}}\rangle + 1\big). \quad (35)$$

The advantage here is that no broadening factor is needed, which is usually required when the δ distribution is taken as a Lorentzian in the FGR rate expression. Moreover, the Lorentzian broadening procedure appears numerically less robust and more sensitive at the low temperatures for tunneling rates and is therefore not further considered here. We finally note that the continuum expressions (34) and (35) can be seen as the

more fundamental form of the FGR, for cases with a continuum of final states, and Eqs. (29) and (30) are discretized versions of this.

The thermal (Boltzmann-weighted) rate constant for transitions originating in initial ("O-bound") state $|m\rangle$ is then given by

$$k_m(T) = P_m(T) \sum_n k_{mn} \,. \tag{36}$$

Here, $P_m(T) = \frac{e^{-\beta(E_m - E_0)}}{\sum_l e^{-\beta(E_l - E_0)}}$ is the Boltzmann weight of the "O-bound" state, $\beta = (k_B T)^{-1}$ and E_0 is the zero-point energy in the "O-bound" well. Also, l are indices of "O-bound" states only. (We assume that the "O-bound" molecule has equilibrated.) The $\sum k_{mn}$ in Eq. (36) includes the sum of both the upward and downward transition rates, calculated in Eqs. (34) and (35), from an initial "O-bound" state with energy E_m, so that transitions to all final C-bound levels up to the classical isomerization barrier, which can be coupled via the phonon bath (up to the Debye frequency), are considered.

In a final step, the total temperature-dependent rate for transitions from "O-bound" to "C-bound" states is calculated by taking the summation of the rates for all "O-bound" states:

$$k(T) = \sum_m k_m(T) \,. \tag{37}$$

At $T = 0$ K, only downward transitions are possible, because $\langle n_b \rangle = 0$, i.e.

$$k_{mn}^{\downarrow}(0) = \frac{2\varepsilon}{\hbar} |V_{mn}|^2 \rho(\omega_{nm}) \tag{38}$$

$$k_{mn}^{\uparrow}(0) = 0 \tag{39}$$

The zero-temperature ground state tunneling rates obtained in this way are contrasted in Table 1 of the main text with corresponding semiclassical WKB rates. In Table S2, the trends in Arrhenius parameters derived both from FGR and WKB (and also TST) are compared. It must be noted that a one-to-one comparison cannot be made because of the different approximations underlying the WKB and FGR models and the different dimensionality of the tunneling path, and also as we will see below, due to the temperature range considered for obtaining the Arrhenius parameters in both cases. Qualitative differences are striking, however.

In practice, ε absorbed the partition function Q giving a modified $\varepsilon' = \varepsilon/Q$. (The weak T-dependence of Q was neglected.) We set $\varepsilon' = 1 \times 10^{-12}$ atomic units throughout. This choice of ε' gives reasonable thermally activated rates when compared to experiment. For ground state tunneling, the rate is 2.6×10^{-5} s^{-1} for the heaviest isotopologue, $^{13}C^{18}O$. The experimental upper limit for this isotope for the buried (overlayer) system is 3×10^{-8} s^{-1} (see section S7). Since our model system is free of CO overlayers, the tunneling rate is expected to be faster compared to the overlayer system.

v. Limitations of the FGR Model

We would like to emphasize that the FGR model developed for this work is highly simplified. For example, unlike the experimentally-studied buried monolayer samples; the theoretical FGR model has no overlayers. The presence of overlayers would restrict motion of the CO adsorbate in the Z direction, which would of course strongly affect the overall isomerization rate. This is why we do not aim to compare the absolute FGR and experimental rates for isomerization, but rather, demonstrate that the large non-intuitive isotope effects observed experimentally could be explained by a state-specific tunneling picture.

Other important approximations represent directions for future work and are summarized as follows:

(i) The spectral density treats every phonon mode as being essentially equally capable to couple to both system modes. Of course, in reality different phonons will couple differently to the individual system modes, which could strongly impact the overall tunneling rates.

(ii) No two-phonon and higher contributions to rates are included, excluding therefore also elastic scattering events which could arise from absorption and emission of the same phonon, connecting initial and final system states of the same energy, i.e., connect pairs of degenerate system states. However, note that the DOS used in our bath model is not zero-valued near zero frequency owing to the frustrated motions of neighboring CO molecules. Hence nearly degenerate states can couple and give rise to non-negligible rates in our model.

(iii) A bilinear coupling model, Eqs. (24) and (25), was used, which is certainly only approximate along the θ coordinate due to the asymmetric double-well potential along this coordinate.

(iv) The system states are considered only in 2D.

Despite these and further simplifications, our minimal model already accounts for the qualitative effects seen in experiment. Specifically, it predicts: an Arrhenius dependence of tunneling rates with effective activation energies lower than the barrier height for a bare monolayer; the occurrence of (few) "tunneling gateways" which dominate the process; and large isotope effects with non-intuitive mass dependence. Specific results (beyond those in the main text) are described in the next subsection. As noted earlier, the results discussed below are restricted to the isotopologues studied experimentally, though the same treatment was also applied to $^{12}C^{18}O$.

b. Results

i. Arrhenius Parameters

We performed FGR total rate calculations for all three isotopologues from 5-31 K. See Fig. 2c of the main text. Two regimes are seen: a deep tunneling regime (T < 20 K), where rates are only weakly dependent on T, and a thermally activated regime exhibiting Arrhenius behavior. As in experiment, the mass-ordering of rates is counter-intuitive both in the deep tunneling and in the activated tunneling regimes. The FGR model shows an activated tunneling regime that is shifted slightly to higher temperatures compared to experiment. For this reason, we compare Arrhenius values taken from a shifted temperature range of 26.8-31 K (see table S2 and inset in Fig. 2c). This chosen range is 4.2 K wide, comparable to experiment. The shift of our activated tunneling regime and therefore also the onset of deep tunneling regime to higher temperatures relative to experiment can be understood by the fact that our theoretical model is for a non-buried adsorbate layer (at a coverage of 1/4). The experiment, on the other hand, probes a buried adsorbate with a higher expected barrier (due to restricted motion) and therefore reduced tunneling.

Of course, quantitative deviations between experiment and our simple model emerge. Most notably, the mass-ordering of activation energies differs; whereas E_a^{FGR} for $^{12}C^{16}O$ is 28 and 52 cm^{-1} below those of $^{13}C^{16}O$ and $^{13}C^{18}O$, respectively, the experimentally-derived result for the lightest isotopologue lies between the two heavier ones. However, as demonstrated by Figure 2b, the correlation between Arrhenius parameters is captured by the FGR results. Finally, while the FGR model captures qualitatively the fact that

heavier particles can tunnel faster than lighter ones, the precise ordering of isotopologues seen in experiment is not reproduced. This is not surprising given the simplicity of the model.

ii. Importance of Tunneling Gateways

State-to-state $k_{mn}(T)$ and initial state-selected $k_m(T)$ isomerization rate constants were calculated for all three experimentally studied isotopologues according to Eqs. (34), (35) and (36). The dominance of tunneling gateways for the heaviest isotopologue, $^{13}C^{18}O$, at T=27K is demonstrated in Fig. 3a of the main text, by plotting the state-to-state breakdown of total rates. In Fig. S12, we present similar state-to-state breakdowns of total rates for all three experimentally studied isotopologues at T = 27 and 30 K. These plots show that tunneling gateways are important for all three isotopologues. For thermally activated gateway tunneling, upward transitions (i.e. absorption of a phonon) are most important. As the temperature is reduced, tunneling involving phonon emission is also seen to become more important. For all three isotopologues, the upward/downward transitions mostly occur via phonon absorption/emission of phonons with energy < 40 cm^{-1}.

Fig. S13 shows initial-state selected rate constants for 27 and 30 K, scaled to the to the total thermal rate constant. Notice that initial O-bound states make transitions to specific C-bound states. The ground state gateway is present in all three isomers and can been seen at the lowest values of E_m. Thermally activated tunneling occurs through specific gateways at higher values of E_m. Fig. S14 shows which product states are involved in gateways and compares upward vs. downward rates constants. Representative wavefunctions involved in tunneling gateways are shown in Fig. S15—also see Fig. 3b&c of the main text. States localized around $\theta = \pm 180°$ (upper panels) correspond to "O-bound" and states around $\theta = 0°$ (lower panels) to "C-bound" configurations. We emphasize that only states with a high degree of excitation along the direction of hindered rotation participate in gateways.

Gateways are also important for deep tunneling at low T. These are evident in Fig. S16 as sticks near E_m=700 cm^{-1}, in particular tor $^{12}C^{16}O$ and $^{13}C^{16}O$. These gateways act as leaky holes through the barrier, leading to a dramatically weaker T-dependence than seen for WKB tunneling and a non-intuitive mass dependence.

iii. Convergence of System Wavefunctions and Ground State Tunneling Rate

The calculation of initial and final-state wavefunctions $\psi_m(\theta, Z)$ and $\psi_n(\theta, Z)$ and corresponding tunneling matrix elements turned out to be a numerical challenge, because the latter depend sensitively on the grid chosen to calculate and represent the wavefunction. In this work, we systematically increased the grid size of our basis for evaluation of the 2D eigenfunctions in order to obtain converged tunneling matrix elements, V_{mn}, for the computation of transition rates. The different grid sizes used are shown in Fig. S17.

Figures S18 through S22 show results of our convergence study for system state energies and FGR matrix elements for selected tunneling gateways. Notice that the energies of the states must be converged to far better than 1 cm^{-1} to obtain converged matrix elements. Without this level of convergence problems arise. For example, Fig. S18(b) shows that at a grid size ($N_Z * N_\theta$) of ~500,000, states 109 and 108 are nearly perfectly degenerate and in an unconverged calculation are artificially mixed with one another. This increase the matrix element for 94,108 by more than an order of magnitude. When the grid size is increased, states 109 and 108 are no longer mixed and this strongly influences the 94,108 matrix element. It is remarkable that the converged energies of states 109 and 108 have only been improved by ~0.5 cm^{-1}, but that this reduces the 94,108 FGR matrix elements by more than a factor of 10. Contrasting this behavior, Fig. S19 demonstrates the convergence relevant to the 132,148 gateway for $^{12}C^{16}O$, which proceeds smoothly with increasing grid size. In Figs. S20 and S21, we show convergence relevant to two gateways present for thermally activated $^{13}C^{18}O$ tunneling. Both gateways involve wave functions that are partially delocalized. Here, the delocalization is consistent for all grid sizes, in contrast to the behavior seen for $^{12}C^{16}O$ states 109 and 108 above. Ground-state tunneling matrix elements and rates are usually found to converge quickly, as demonstrated for the latter and $^{13}C^{18}O$ in Fig. S22.

S7. Attempted Measurement of the Ground State Tunneling Lifetime for $^{13}C^{18}O$

To determine the rate of ground-state flipping from O-down to C-down, the O-down sample was prepared as described in section S1 at a surface temperature of 7K and monitored for about 120 hours (Fig. S23). The change in integrated absorbance within this time is only ~3%. Based on this, we calculate a lifetime on the order of 1 year, which should be considered a lower limit.

S8. Supplementary Figures

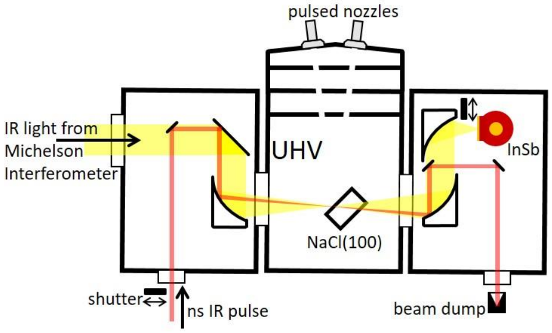

Figure S1. Experimental setup. The NaCl(100) crystal is attached to a 4D translation stage within the UHV chamber. The sample is moved between the position for pulsed molecular beam dosing and laser excitation/FTIR spectral acquisition.

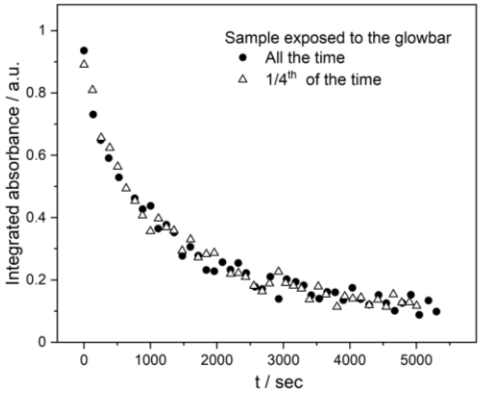

Figure S2: Effect of FTIR glow bar light on the decay of the O-down population. Kinetic traces of O-down population (see section S2) in a buried $^{13}C^{18}O$ monolayer at 21.5K following laser excitation of the $^{12}C^{16}O$ overlayer at 2138.6 cm^{-1}. Solid circles correspond to a sample that was exposed to a 4 times higher glow bar light dose compared to the sample represented by the open triangles.

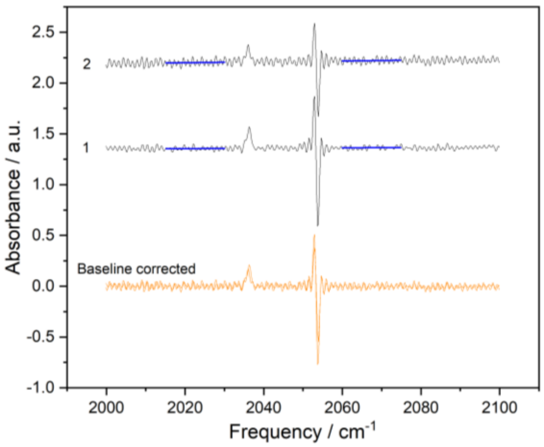

Figure S3: Illustration of baseline correction of FTIR spectra. The raw spectra (black) correspond to a buried $^{13}C^{18}O$ monolayer following excitation of the $^{12}C^{16}O$ overlayer at 2138.6 cm^{-1}, where spectrum 2 was obtained at a later time than spectrum 1. The thick blue lines denote the range of the fitted straight line and the two background-subtracted spectra are shown in orange.

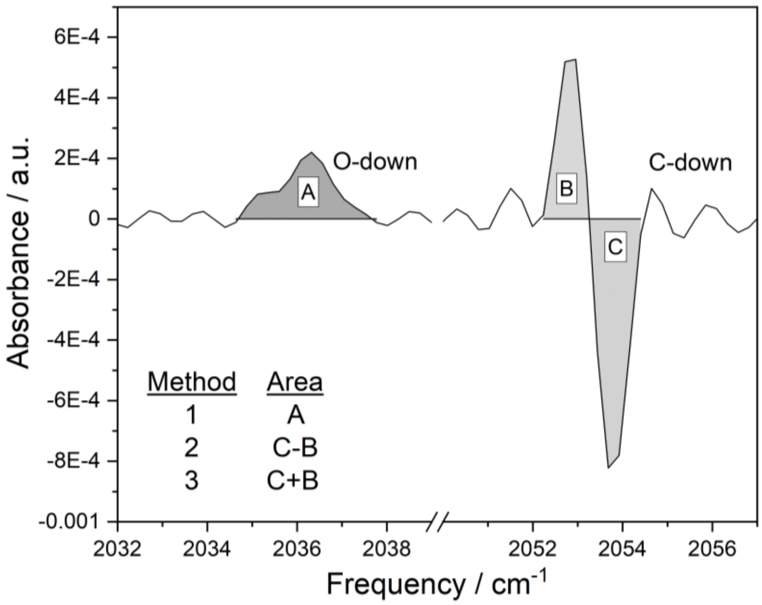

Figure S4: Illustration of methods for determining the relative population of O-down $^{13}C^{18}O$ from the difference absorbance spectra. For methods 1 and 2 integrals over the O-down (area A) and C-down feature (area C – B) are calculated, respectively. For method 3 the area B + C is evaluated.

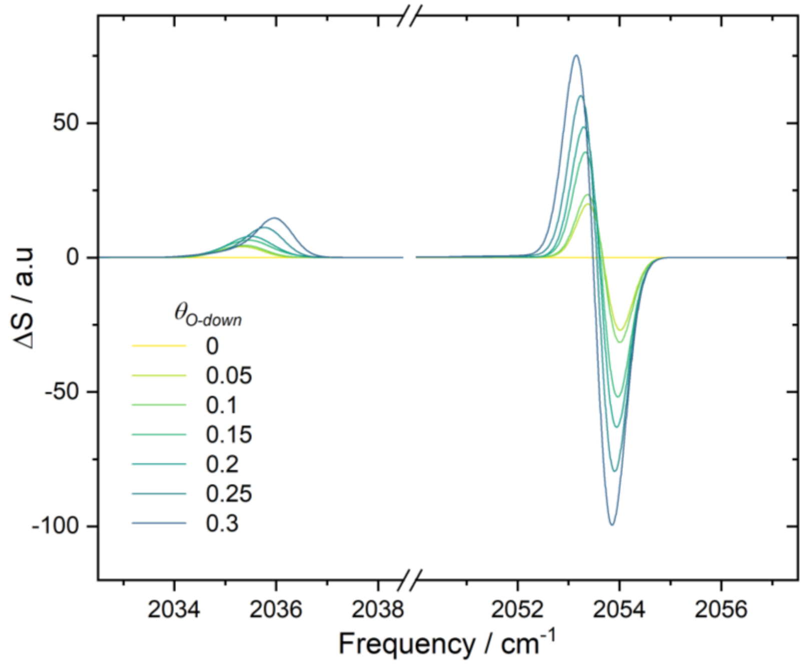

Figure S5: Difference absorbance spectra derived from the exciton model. $\Delta S(\tilde{\nu})$ is calculated for various fractions of O-down population (θ_{O-down}), where the positions of the O-down molecules are chosen randomly in a 30×30 grid. For these spectra, the chosen $hc\tilde{\nu}_0$ values were those of the $^{13}C^{18}O$ monolayer isomers, so that these results may be compared to experimental spectra obtained following overlayer excitation in a buried $^{13}C^{18}O$ monolayer sample

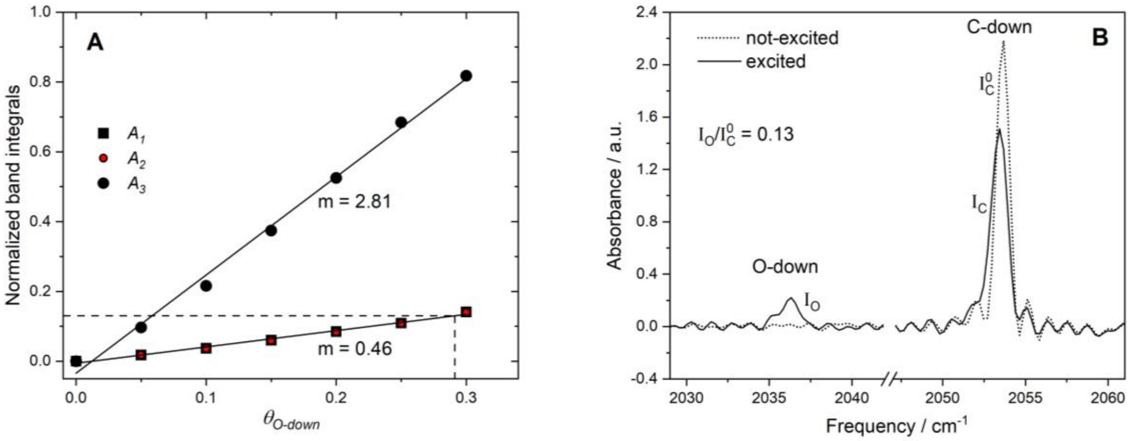

Figure S6: Comparison of different analysis methods for determining the coverage of O-down $^{13}C^{18}O$ molecules. (A) Relation between the simulated integrated absorbance and the fraction (θ_{O-down}) of molecules flipped in the exciton model. The integrated band absorbances (A_{1-3} as defined above) are normalized to the integral of the C-down peak in the unexcited sample (I_C^0). **(B)** Experimental absorbance spectra prior to (dotted line) and immediately after (solid line) laser excitation from a typical lifetime measurement. Given the I_O/I_C^0 value of 0.13, we may use the results of method 1 to estimate from panel (A) that ~30% of the molecules are flipped (dashed lines).

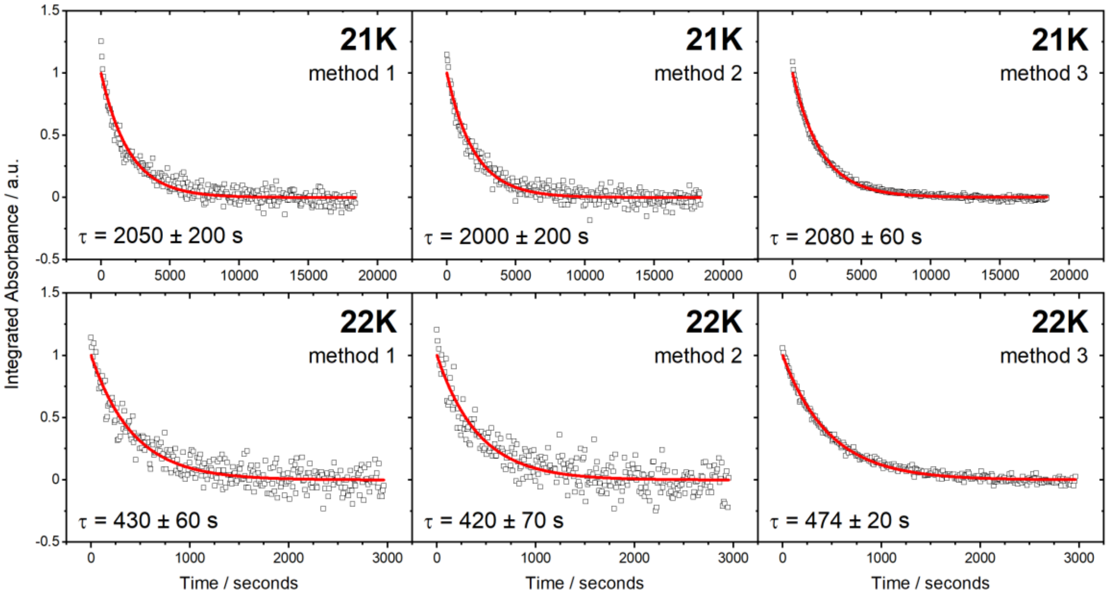

Figure S7. Comparison between the integrated absorbances obtained from the different analysis methods. Methods 1 (left), 2 (middle) and 3 (right) are applied to the data obtained from a $^{13}C^{18}O$ monolayer sample covered by $^{12}C^{16}O$ at 21 K (top) and 22 K (bottom). The red lines show the fitted exponential decays, and the resultant decay constants are also shown. The error is calculated with 95% confidence interval. Note that the kinetic traces shown here correspond to a single measurement; the Arrhenius plot (Fig. 2a) and table S1 show the average of values obtained from several such experiments.

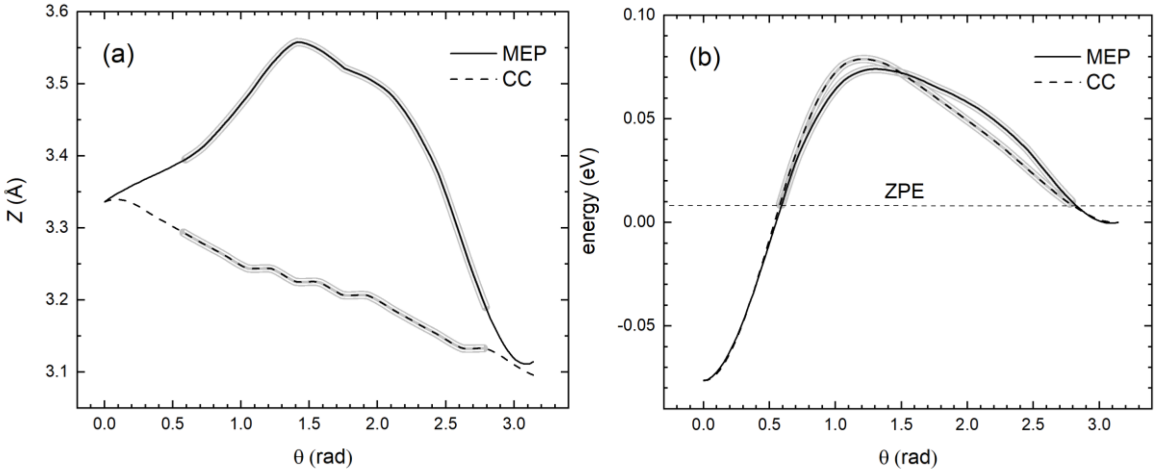

Figure S8: WKB tunneling pathways. Panel (a) shows the minimum energy path (MEP) and the corner cutting (CC) path used for the WKB calculations. Panel (b) shows the corresponding energy profiles along the two paths. The energy is relative to the classical O-down minimum. The region of integration between turning points is shown as thickened grey line for ground state tunneling of the $^{12}C^{16}O$, whose ZPE is also indicated.

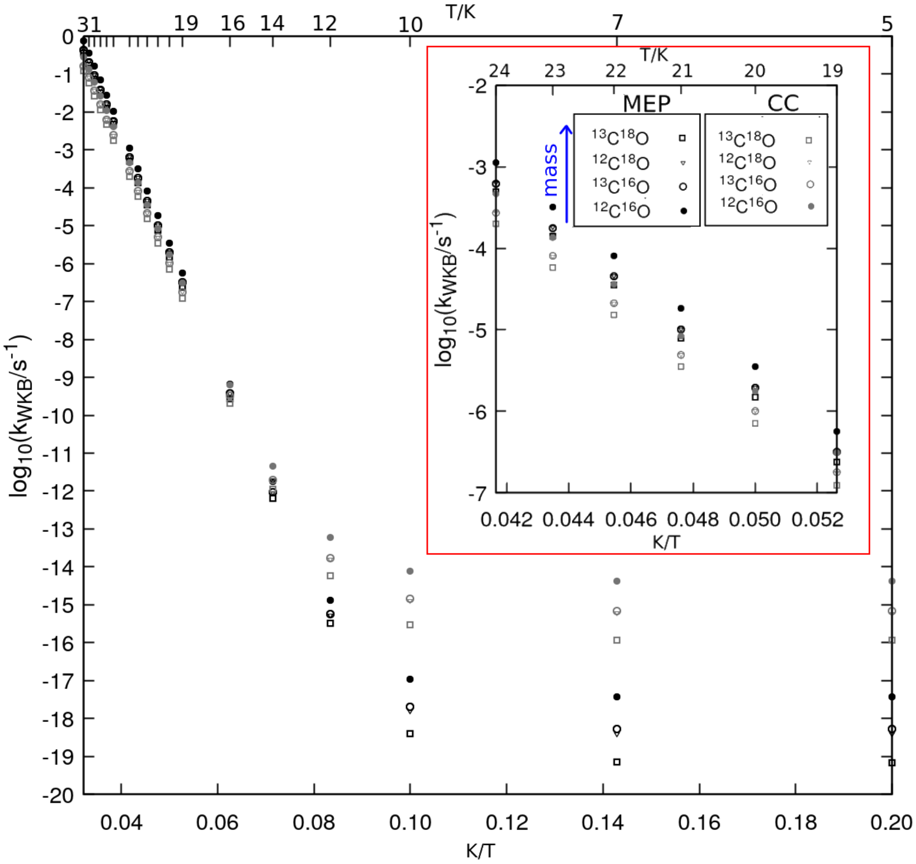

Figure S9: Thermally activated WKB tunneling rates for four isotopologues, $^{12}C^{16}O$, $^{13}C^{16}O$, $^{12}C^{18}O$, and $^{13}C^{18}O$. The rate constants are shown as base-10 logarithms. The inset shows the calculated rates in the experimental temperature regime [19-24] K which was fitted to obtain Arrhenius parameters. MEP and CC indicates the use of the minimum energy path or the corner-cutting path, respectively, shown in Fig. S8.

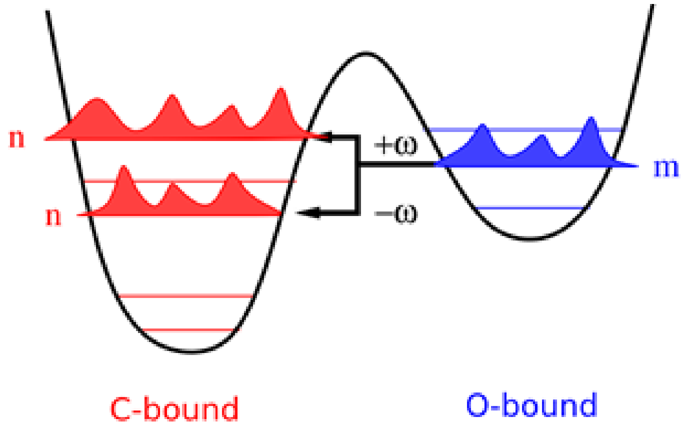

Figure S10. Schematic picture of phonon-mediated resonant tunneling. A vibrational state, denoted as m, that is localized in the "O-bound" well of the potential can reach a higher-lying state n in the "C-bound" well by either relaxing / absorbing a surface phonon with frequency ω $(+)$, leading to an upward rate $k^{\uparrow}_{mn}$ (see the corresponding FGR expression later), or a lower-lying state n by exciting / emitting a surface phonon with (possibly another) frequency ω $(-)$, leading to a downward rate $k^{\downarrow}_{mn}$. Boltzmann averaging over all possible initial states m and summing over all possible final states n gives a T-dependent, total isomerization rate which is strongly isotope dependent.

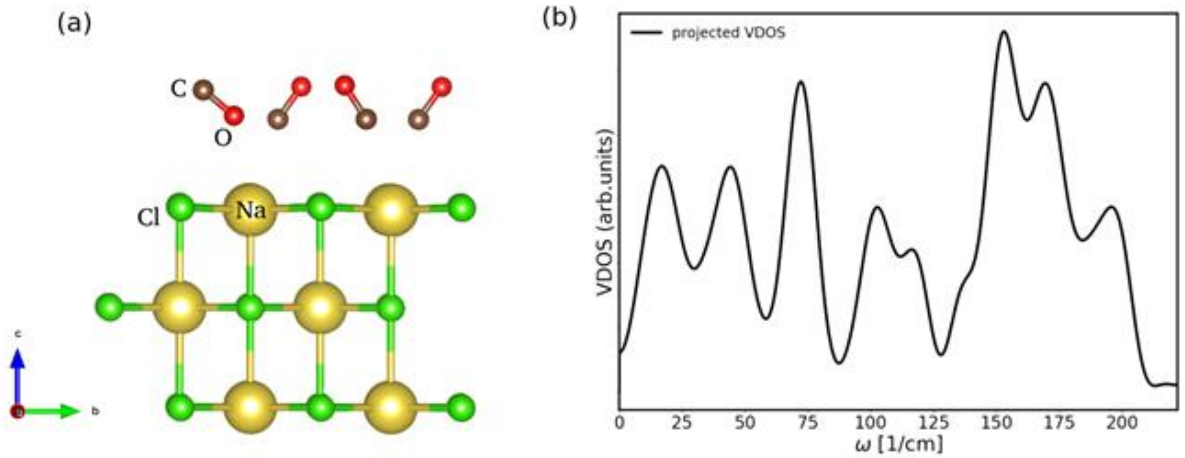

Figure S11. (a) Configuration used to evaluate the projected VDOS via AIMD, with one "O-bound" CO per unit cell (see text). (b) Resulting projected VDOS accounting for the neighboring CO adsorbates and NaCl(100) phonons in 1 ML CO:NaCl(100) at 30K.

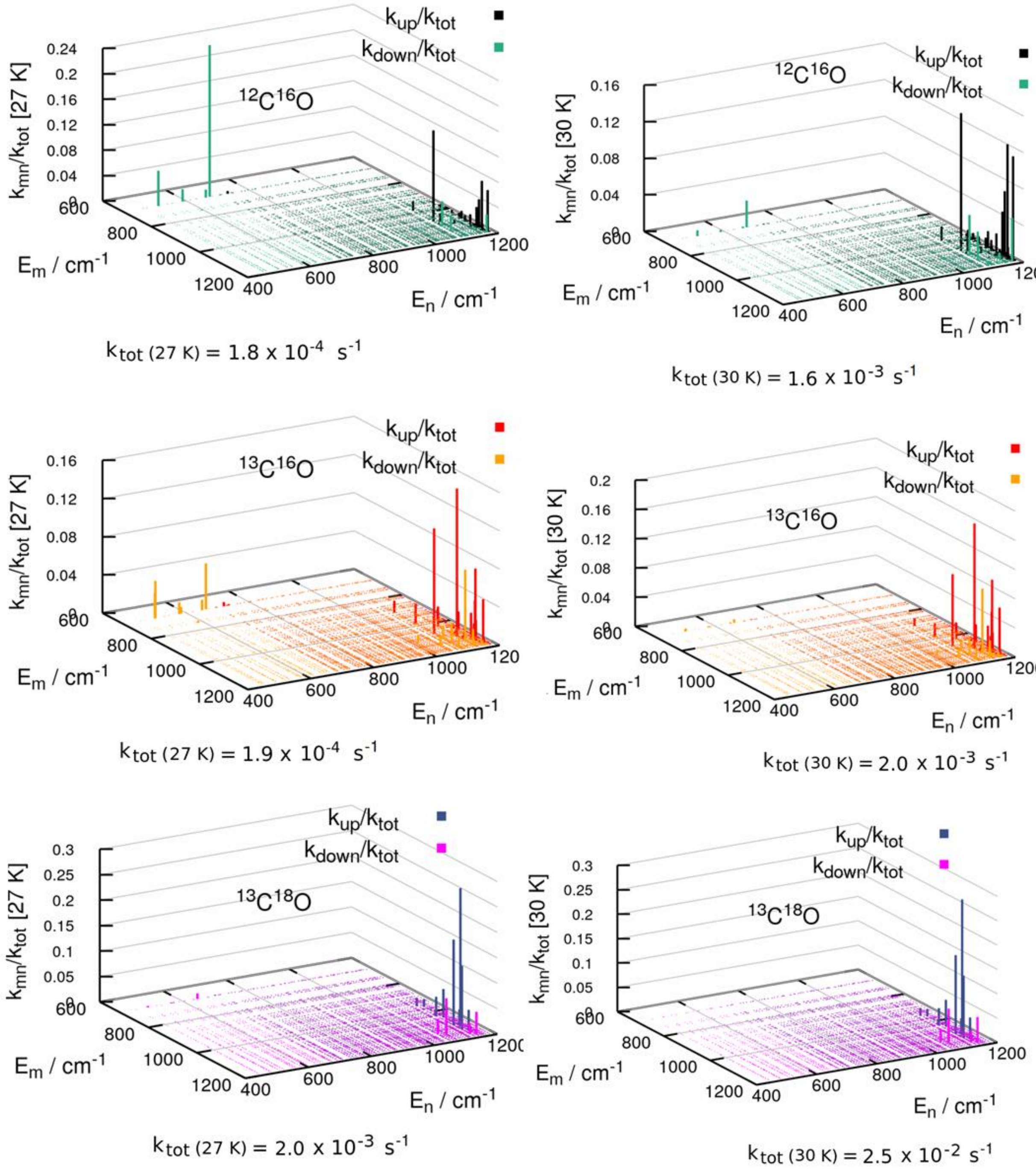

Figure S12. State-to-state breakdown of total FGR rates for three isotopologues at T = 27 K (left) and T = 30 K (right). Each vertical bar indicates the fraction of the total rate represented by the state-to-state upward rate and downward rate (color coded), describing transitions from "O-bound" states at energy E_m to "C-bound" states at E_n. All energies are with respect to the classical minimum of the "C-bound" configuration.

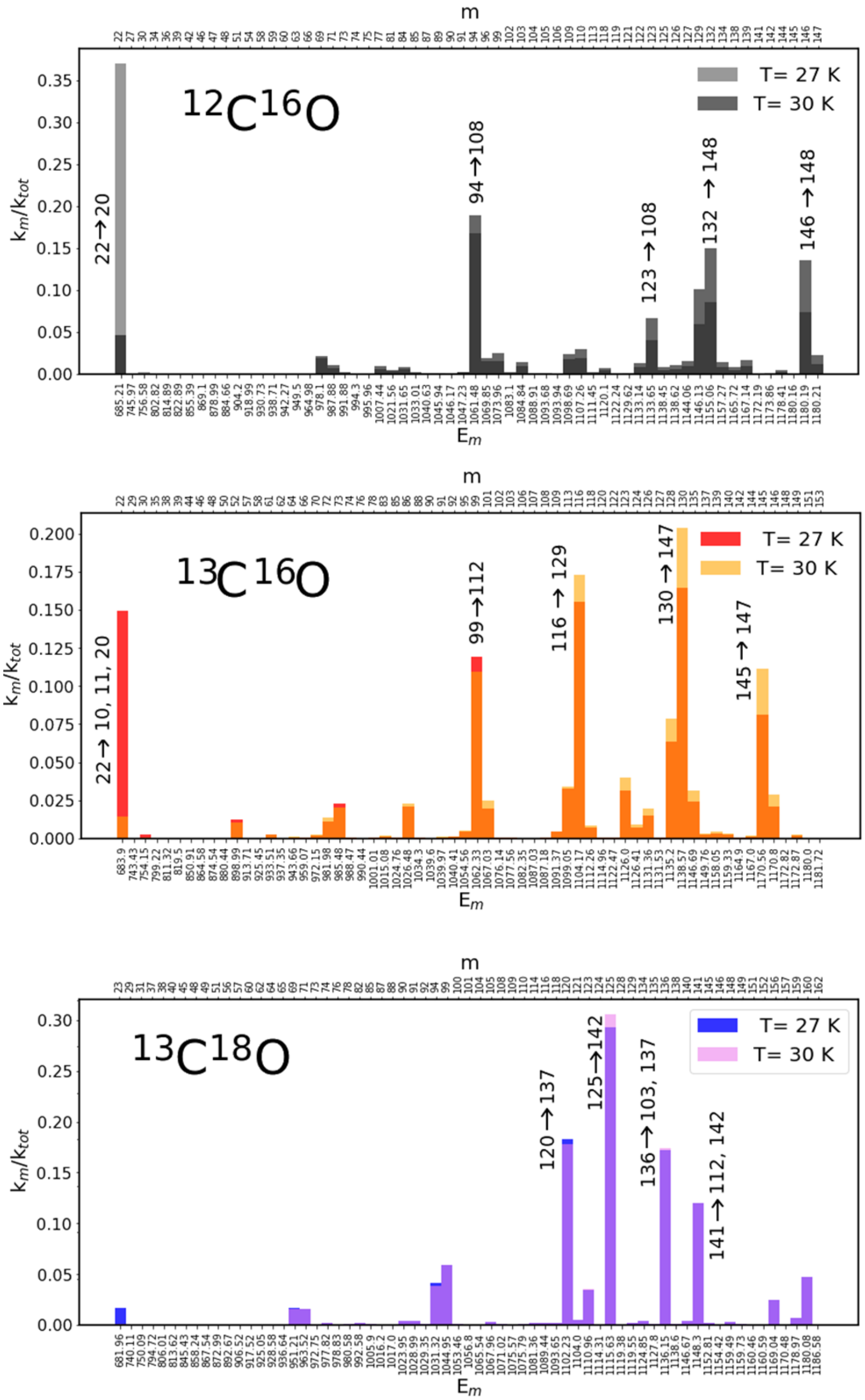

Figure S13. Ratio of initial state-selected (upward plus downward) rates k_m to total rates at T =27, 30 K for ¼ ML **(a)** $^{12}C^{16}O$, **(b)** $^{13}C^{16}O$, **(c)** $^{13}C^{18}O$ on NaCl(100) vs. initial "O-bound" states $|m\rangle$. The numbers on the x-axis give the energies of the initial states $|m\rangle$ relative to the classical minimum of the "C-bound" configuration. The barrier height is 1187 cm^{-1} on this scale and the lowest energy "O-bound" states are at 685, 684 and 682 cm^{-1} for the three isotopologues. Note: States involved in giving dominant contributions to rates have been highlighted in the figure with $m \rightarrow n$. When more than one final state contributes to the initial-state selected rate k_m, it is indicated by $m \rightarrow n_1, n_2, \ldots$ and so on.

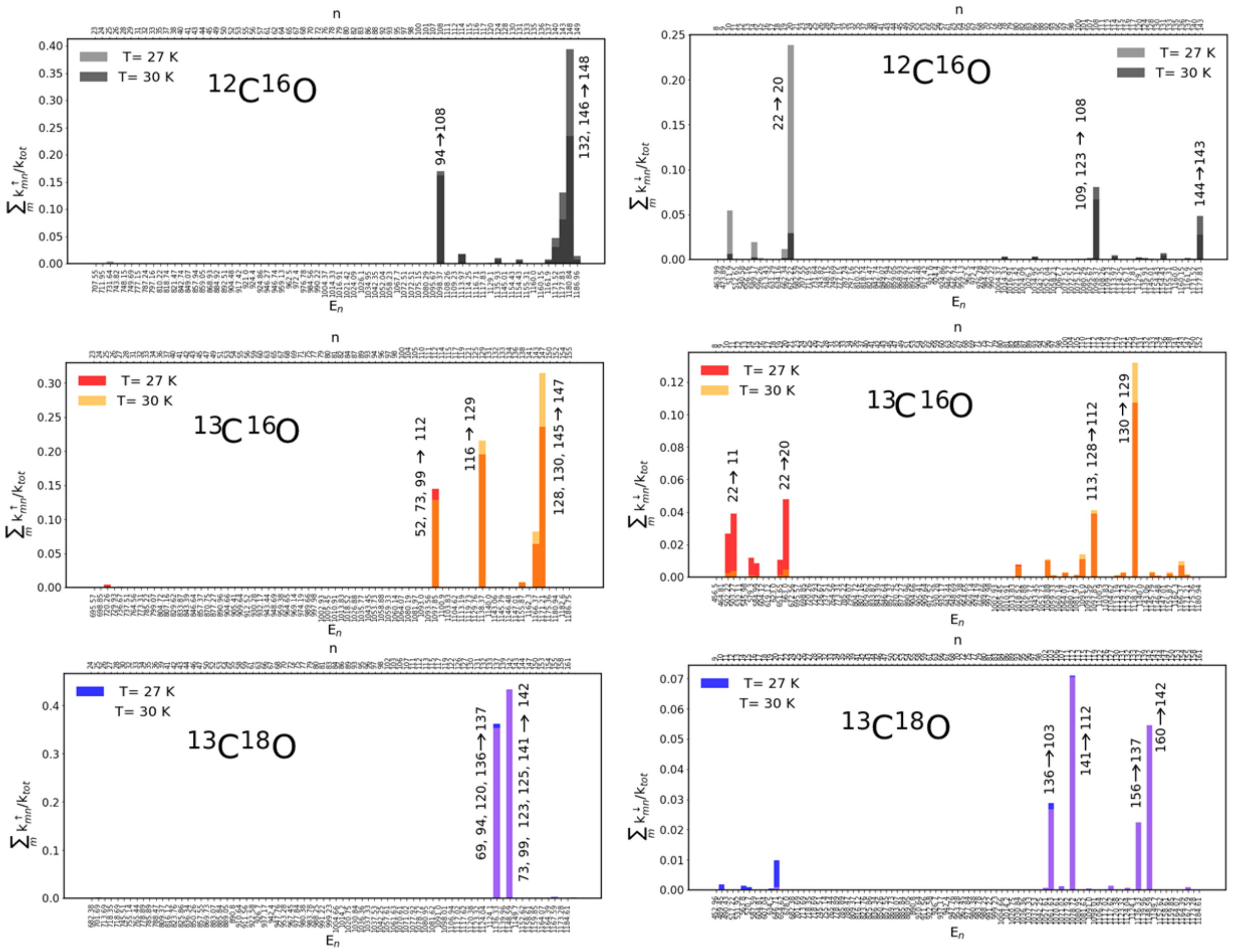

Figure S14. Ratio of final state-selected upward (left panel) and downward (right panel) rates from initial "O-bound" states to the total (upward + downward) rates (i.e., $\sum_m k_{mn}^{\uparrow\downarrow}(\mathrm{T})/k(\mathrm{T})$ at T = 27, 30 K for $^{12}C^{16}O$ (top), $^{13}C^{16}O$ (middle), $^{13}C^{18}O$ (bottom) as a function of final "C-bound" state energies, E_n (energies on the bottom side of the x-axis, state numbers n on the top side of the x-axis). Note: States giving dominant contributions to rates have been highlighted in the figure with $m \rightarrow n$. When more than one initial state contribute to the final-state selected rate, it is indicated by $m_1, m_2, \ldots \rightarrow n$ and so on.

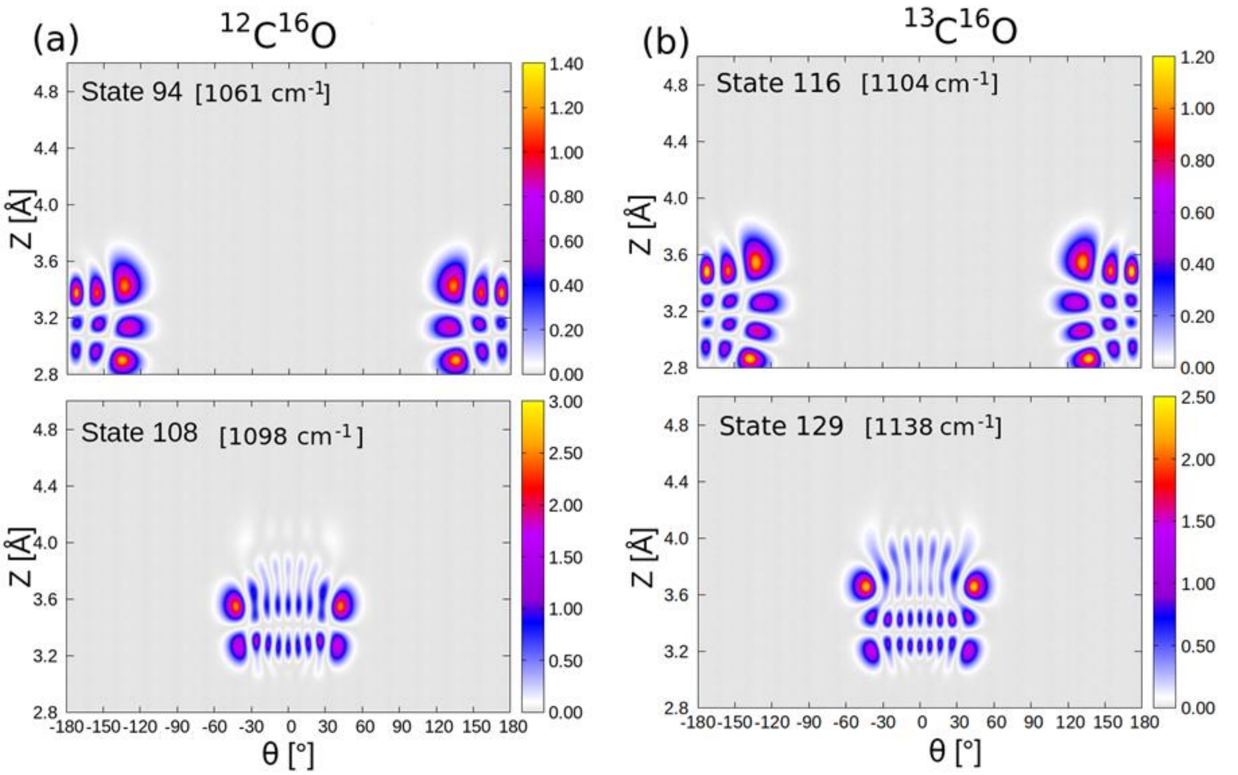

Figure S15. One set of wavefunctions describing the quantum-gateways dominating calculated tunneling rates for (a) $^{12}C^{16}O$, (b) $^{13}C^{16}O$ for T=[27-30] K. In each panel, the angle of CO with respect to the surface normal (θ) is the vertical axis, where θ=0° represents the C-O bond parallel to the surface normal in the "C-bound configuration". The horizontal axis is the distance of the CO molecule from the surface (Z). State energies are given relative to the classical "C-bound" minimum in the PES. State numbers are w.r.t to the "C-bound" ground state being State 0.

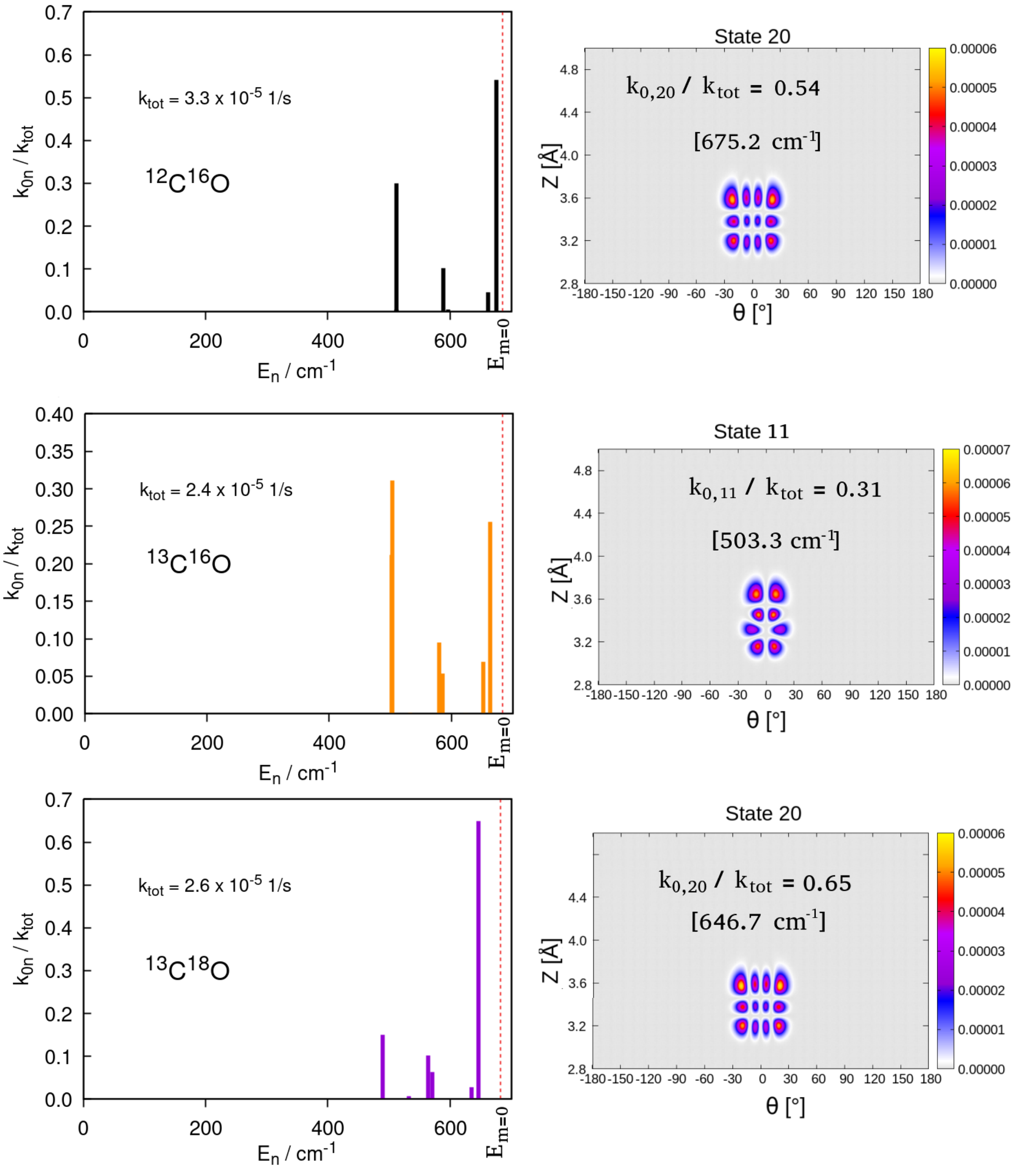

Figure S16. Ground-state tunneling gateways for $^{12}C^{16}O$, $^{13}C^{16}O$, and $^{13}C^{18}O$. The contribution for each C-bound level n shows that the deep-tunneling rates are dominated by population-transfer to a few specific C-bound states. The dashed red line in the left panels indicate $E_{m=0}$, i.e, the energy of the ground state O-bound configuration. For each isotopologue, the wavefunction densities of the most significant C-bound state is shown. All energies are relative to the classical minimum of the C-bound configuration.

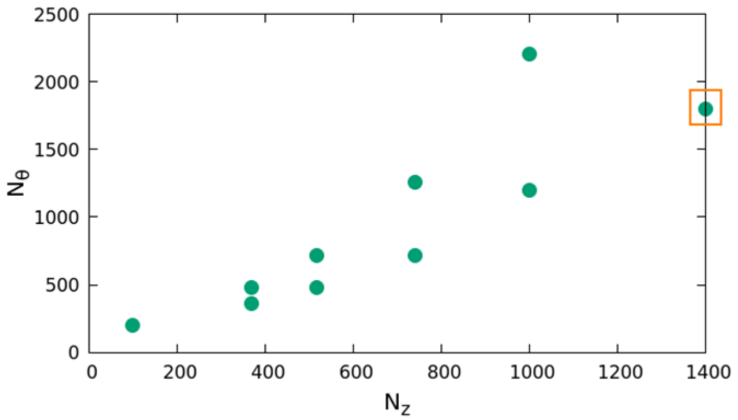

Figure S17. Different grids used for computation of 2D eigenfunctions. The grid size ($N_Z = 1400$, $N_\theta = 1800$ (orange box)) was used for calculating the 2D eigenfunctions used in the rate computation for all three isotopologues discussed above.

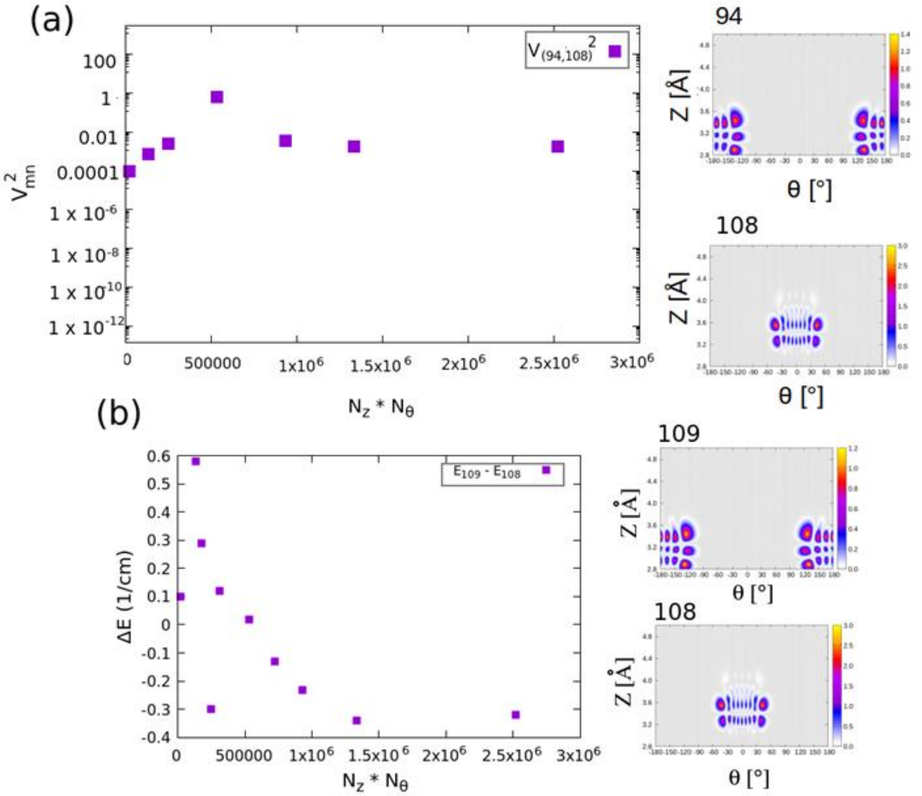

Figure S18. (a) Variation of square of matrix elements for a tunneling gateway pair for $^{12}C^{16}O$ with grid size (in atomic units). (b) Variation in energy difference between one state of the tunneling gateway pair in (a) and an eigenstate present at nearly degenerate energy to it. Corresponding wavefunctions are also shown.

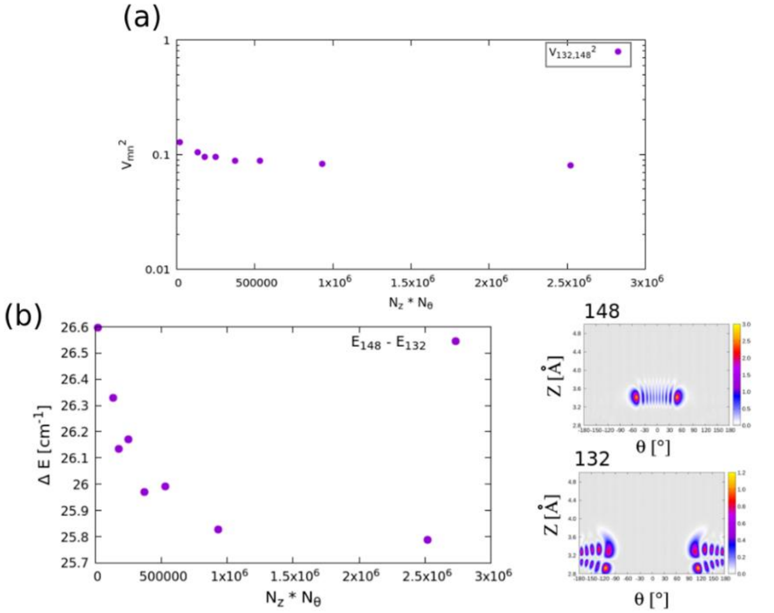

Figure S19. (a) Variation of square of matrix elements for another tunneling gateway pair for $^{12}C^{16}O$ with grid size (in atomic units). (b) Variation in energy difference between those two states, with corresponding wavefunctions.

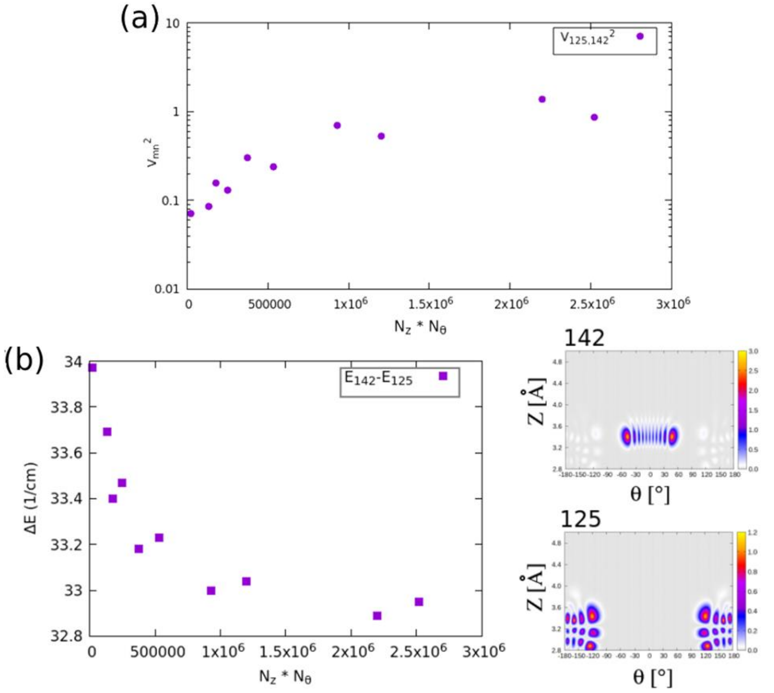

Figure S20. (a) Variation of square of matrix elements for a tunneling gateway pair for $^{13}C^{18}O$ with grid size (in atomic units). (b) Variation in energy difference between those two states, with corresponding wavefunctions.

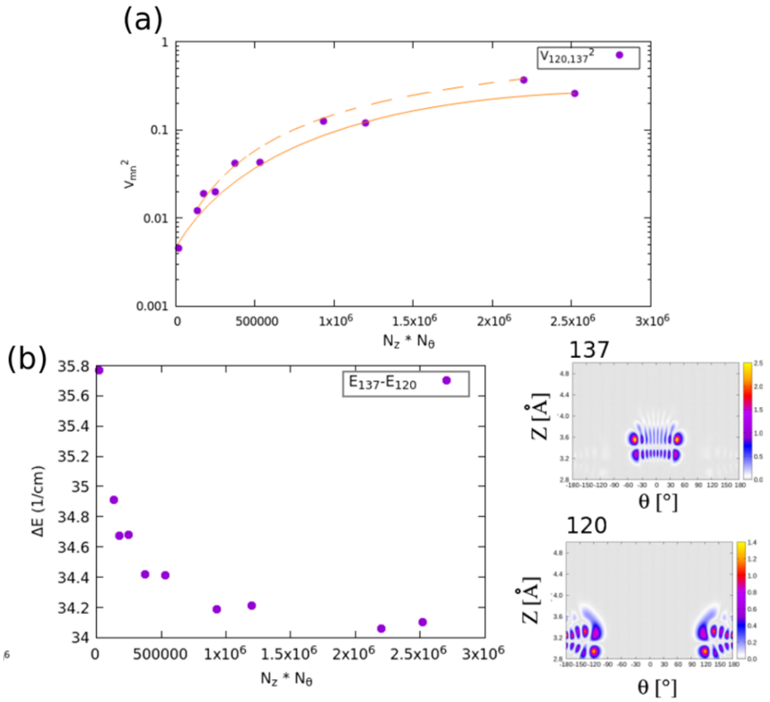

Figure S21. (a) Variation of square of matrix elements for another tunneling gateway pair for $^{13}C^{18}O$ with grid size (in atomic units). (b) Variation in energy difference between those two states, with corresponding wavefunctions. The oscillations in V_{mn}^2 between grid points occur due to change in respective N_Z and N_θ grid points. Orange lines guide the reader's eyes to see convergence trends.

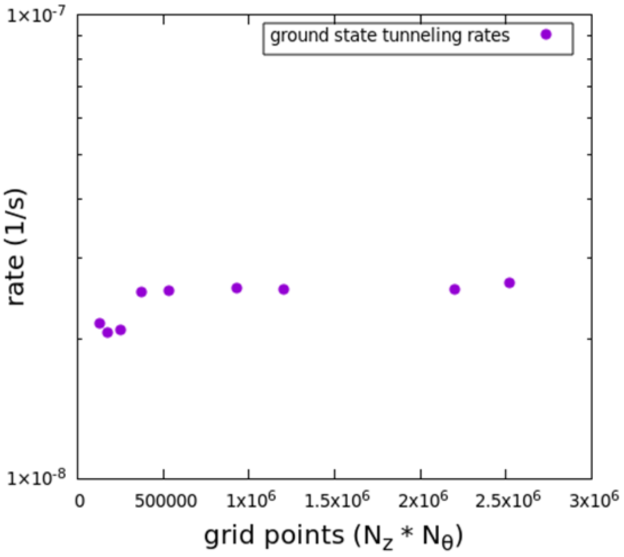

Figure S22. Variation of ground state tunneling rates for $^{13}C^{18}O$ w.r.t grid size.

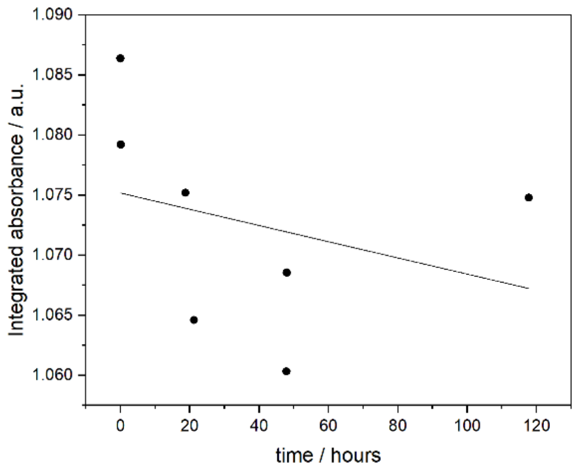

Figure S23: Long time measurement of the absorbance of O-down $^{13}C^{18}O$ at T =7K. The points show results of measurements and the line is a best fit exponential corresponding to 2 year lifetime. As we cannot rule out that this is noise, the measurements are only useful to put a lower limit on the lifetime of the ground state O-down isomer.

S9. References

1 Lau, J. A. *et al.* Observation of an isomerizing double-well quantum system in the condensed phase. **367**, 175-178 (2020).

2 Chang, H. C., Richardson, H. H. & Ewing, G. E. Epitaxial growth of CO on NaCl(100) studied by infrared spectroscopy. *J Chem Phys* **89**, 7561 (1988).

3 Lau, J. A. *et al.* Transporting and concentrating vibrational energy to promote isomerization. **589**, 391-395 (2021).

4 Disselkamp, R., Chang, H.-C. & Ewing, G. E. Infrared spectroscopy of CO on NaCl(100) IV. Bandshape analysis. **240**, 193-210 (1990).

5 Dai, D. J. & Ewing, G. E. Vibrational Overtone Spectroscopy and Coupling Effects in Monolayer CO on NaCl(100). *Surf Sci* **312**, 239-249 (1994).

6 Sinha, S. & Saalfrank, P. "Inverted" CO molecules on NaCl(100): A quantum mechanical study. **23**, 7860-7874 (2021).

7 Marcus, R. A. & Coltrin, M. E. A new tunneling path for reactions such as $H+H_2 \rightarrow H_2+H$. **67**, 2609-2613 (1977).

8 Echave, J. & Clary, D. C. Potential Optimized Discrete Variable Representation. *Chem Phys Lett* **190**, 225-230 (1992).

9 Balint-Kurti, G. G., Dixon, R. N. & Marston, C. C. Grid Methods for Solving the Schrodinger-Equation and Time-Dependent Quantum Dynamics of Molecular Photofragmentation and Reactive Scattering Processes. *Int Rev Phys Chem* **11**, 317-344 (1992).

10 Andrianov, I. & Saalfrank, P. Theoretical study of vibration-phonon coupling of H adsorbed on a Si(100) surface. **124**, 034710 (2006).

11 Hughes, K. H., Christ, C. D. & Burghardt, I. Effective-mode representation of non-Markovian dynamics: A hierarchical approximation of the spectral density. I. Application to single surface dynamics. **131**, 024109 (2009).

12 Melani, G., Nagata, Y., Campen, R. K. & Saalfrank, P. Vibrational spectra of dissoiatively adsorbed D_2O on Al-terminated Al_2O_3 (0001) surfaces from *ab initio* molecular dynamics. **150**, 244701 (2019).

13 Lorenz, U. & Saalfrank, P. A novel system-bath Hamiltonian for vibration-phonon coupling: Formulation, and application to the relaxation of Si-H and Si-D bending modes of H/D:Si(100)-(2x1). **482**, 69 (2017).
